# Improved conditioning for hematopoietic chimerism induces islet tolerance to cure diabetes

**DOI:** 10.1172/jci.insight.194491

**Published:** 2026-04-09

**Authors:** Stephan A. Ramos, Preksha Bhagchandani, Diego M. Burgos, Xueying Gu, Richard Rodriguez, Nadia Nourin, Martin Neukam, Shiva Pathak, Judith A. Shizuru, Seung K. Kim

**Affiliations:** 1 Department of Developmental Biology,; 2 Division of Blood and Marrow Transplantation, Department of Medicine,; 3 Stanford Diabetes Research Center, and; 4 Northern California Breakthrough T1D Center of Excellence, Stanford School of Medicine, Stanford, California, USA.

**Keywords:** Hematology, Immunology, Bone marrow transplantation, Immunotherapy, Transplantation

## Abstract

Mixed hematopoietic chimerism after hematopoietic cell transplantation (HCT) can modulate the immune system and induce tolerance to allogeneic tissues. However, bone marrow conditioning–related toxicities preclude wider adoption of HCT for transplant allotolerance. We sought agents that reduced conditioning intensity, while promoting durable mixed chimerism after HCT across complete MHC mismatch in diabetic mice, permitting islet allotransplantation and diabetes reversal. We systematically tested baricitinib (JAK1/2 inhibitor), venetoclax (Bcl-2 inhibitor), and CD47 antibody, agents in current clinical use, and quantified hematopoietic chimerism after HCT. Combined with CD117 antibody, transient T cell depletion, and just 10 centigray total body irradiation, these agents enabled durable mixed chimerism and matching alloislet tolerance to cure diabetes without evidence of graft-versus-host disease. Thus, we have developed a conditioning regimen to promote allogeneic mixed hematopoietic chimerism and transplanted islet allotolerance that minimizes conditioning radiation and cures diabetes.

## Abstract 2

We developed a non-toxic conditioning regimen with virtually no radiation that enables stable mixed hematopoietic chimerism and islet allotolerance, curing diabetes in mice without immunosuppression.

## Introduction

Transplantation of MHC-mismatched allogeneic islets is an FDA-approved approach to reverse diabetes after pancreatic β cell loss (1, 2). Though effective for restoring glycemic control, standard clinical practice uses chronic immunosuppression to prevent islet allorejection (3), which is associated with considerable toxicities. These include cell and renal injury, increased risk of opportunistic infections and malignancies, and chronic rejection, morbidities that have limited widespread adoption of islet allotransplantation in diabetes (4). Thus, islet cell replacement strategies have focused intensively on establishing immune allotolerance without systemic immunosuppression.

Hematopoietic stem cells (HSCs) are multipotent stem cells that reside in specialized bone marrow niches and are capable of generating all blood and immune cell lineages (5,6). After HCT, complete hematopoietic and immune reconstitution by donor HSCs can cure life-threatening conditions like blood disorders and malignancies and can correct autoimmunity. Mixed hematopoietic chimerism after HCT, where recipient- and donor-derived HSCs coexist, is an effective method to modulate the recipient immune system and establish tolerance to allogeneic donor-matched tissues and solid organs (7–11). Mixed chimerism can reshape central and peripheral immune tolerance, promoting deletion or suppression of allo- (donor) reactive and auto- (self) reactive immune cells while fostering regulatory immune cell populations that support acceptance of donor-matched allogeneic grafts. Beyond enabling immune tolerance, mixed chimerism can contribute to the suppression of autoreactive immune responses, offering potential therapeutic benefits for autoimmune diseases, like type 1 diabetes (12).

To achieve successful engraftment of donor HSCs and establish mixed hematopoietic chimerism, recipients undergo prior bone marrow “conditioning.” Conditioning is a preparative regimen that creates space in the bone marrow niche for donor HSC engraftment and transiently suppresses the recipient immune system to prevent acute graft rejection (13). However, prior conditioning regimes were largely developed for malignancies and rely on complete or near-complete (myeloablative) eradication of the recipient hematopoietic system with radiation therapy (XRT) and/or genotoxic or cytotoxic chemotherapy. Thus, standard conditioning is associated with severe toxicities, including infections, secondary malignancies, and organ damage, preventing broader adoption of mixed chimerism-based approaches for nonmalignant conditions such as diabetes (14, 15). Mere dose reduction of myeloablative conditioning agents to mitigate conditioning intensity and toxicity unfortunately results in HSC engraftment failure (16). Although multiple agents have been explored to reduce conditioning intensity in preclinical models, clinical translation has been limited owing to systemic toxicity, graft-versus-host disease (GVHD), and inefficacy in humans (12, 17–20). Thus, the development of safer nonmyeloablative (NMA) conditioning regimes could advance and expand use of mixed hematopoietic chimerism for transplant tolerance (21).

Previously, we reported a NMA conditioning strategy incorporating αCD117 antibody and a single 300 centigray (cGy) dose of total body irradiation (TBI) for curing established diabetes after allogeneic HCT and islet transplantation (22). However, this TBI dose is associated with increased risk for secondary malignancy and infertility, precluding its use in patient subsets, including children and women of childbearing age (22, 23).

A promising strategy to achieve effective, less toxic conditioning involves targeting specific cellular pathways involved in immune regulation and hematopoietic niche remodeling (24, 25). Baricitinib, a Janus kinase 1/2 (JAK1/2) inhibitor, modulates immune responses by reducing inflammatory cytokine signaling and attenuating T and NK cell responses and enhancing engraftment (25, 26). CD47 is a transmembrane protein expressed on many cell types, including HSCs, that interacts with SIRP on neutrophils and macrophages to prevent antibody-dependent cell-mediated cytotoxicity and phagocytosis (27, 28). Used along side other conditioning agents, CD47 antibody therapy disrupts this “don’t-eat-me” signal on HSCs, facilitating their clearance from the bone marrow niche (29, 30). Venetoclax is a Bcl-2 inhibitor that selectively induces apoptosis in hematopoietic and immune cells while sparing nontarget tissues and promotes donor HSC engraftment with reduced XRT (31). These clinically portable, nongenotoxic agents act on distinct pathways to support donor cell engraftment while reducing conditioning toxicity. Here, when combined with CD117 antibody, transient T cell depletion, and only 10 cGy TBI, these agents enabled durable mixed hematopoietic chimerism and matching alloislet tolerance to cure established diabetes without evidence of GVHD. Thus, we present a clinically relevant, NMA conditioning regimen to promote allogeneic mixed hematopoietic chimerism and transplanted islet allotolerance for diabetes.

## Results

### JAK1/2 inhibition permits reduced radiation in hematopoietic conditioning to achieve durable mixed chimerism.

**Figure 1:**
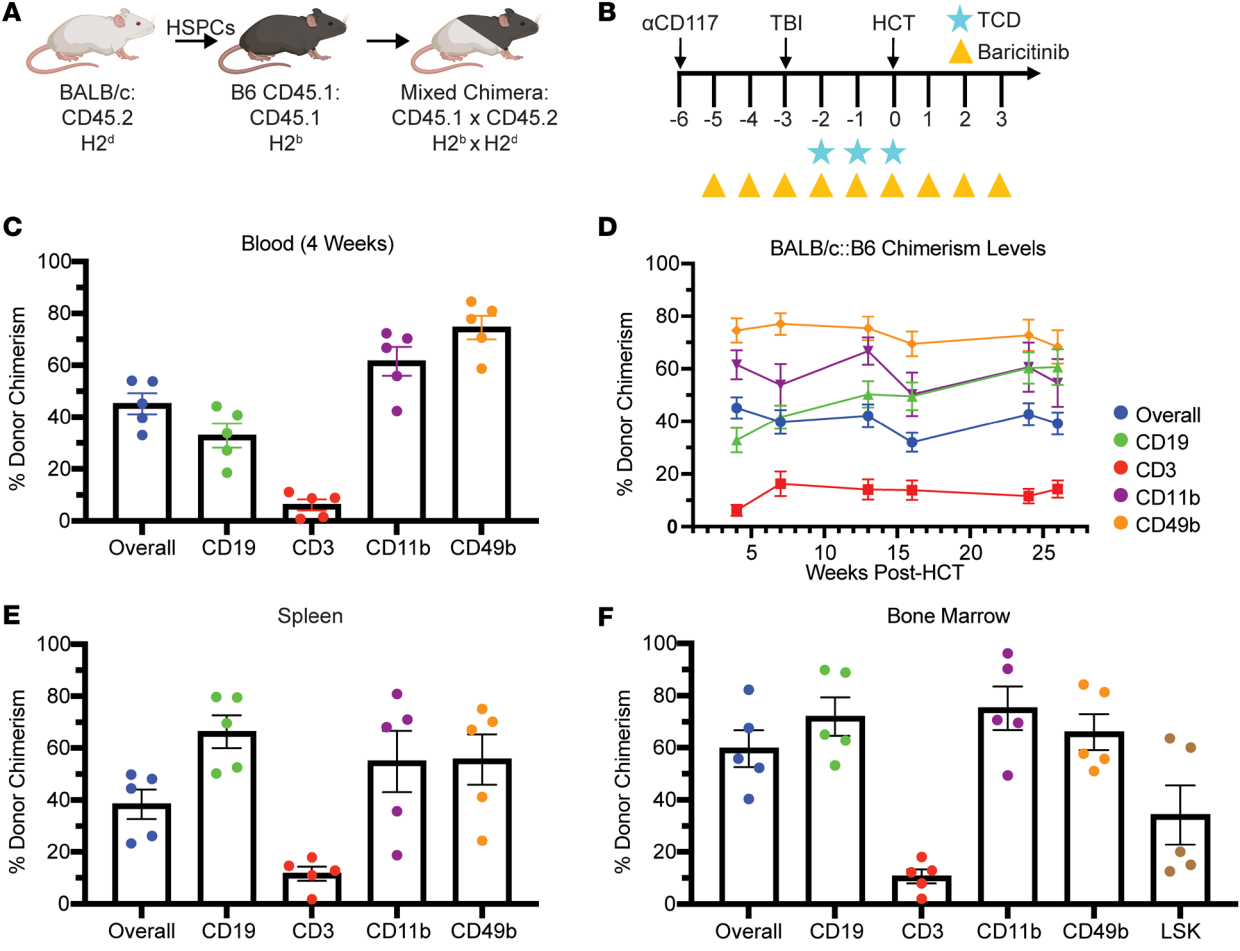
Nonmyeloablative conditioning with baricitinib and 75 cGy TBI permits durable allogeneic donor chimerism. (**A**) Transplantation model and strains used. (**B**) Experimental conditioning and transplantation timeline. (**C**) Multilineage chimerism analysis of peripheral blood 4 weeks after HCT. (**D**) Longitudinal multilineage chimerism analysis of peripheral blood through 26 weeks after HCT. (**E**) Multilineage chimerism analysis of host spleen 26 weeks after HCT. (**F**) Multilineage chimerism analysis, including Lin^–^SCA1^+^cKIT^+^ (LSK) HSCs, of host bone marrow 26 weeks after HCT. *n* = 5 animals, from 1 experiment. Data are presented as mean ± SEM. TBI, total body irradiation; HCT, hematopoietic cell transplant; TCD, T cell depletion. See also Supplemental Figure 1.

A prior NMA conditioning regimen we developed uses CD117 antibody, transient T cell depletion (hereafter referred to as TCD) with αCD4 and αCD8 antibodies, and 200–300 cGy TBI to establish durable mixed hematopoietic chimerism after HCT with MHC-mismatched bone marrow (22). To improve conditioning further, we sought reagents with established clinical safety that would permit reduced radiation dosage. Baricitinib is an immunosuppressive agent that inhibits NK and T cells via JAK1/2 blockade and is well-tolerated in studies of allogeneic HSC engraftment (26, 32). To evaluate if baricitinib could reduce conditioning TBI intensity, nondiabetic B6 CD45.1^+^ mice were administered baricitinib (400 g/d) from days –5 to day +3 and TCD from day –2 to day 0, irradiated with deescalated doses of 75, 50, or 25 cGy TBI on day –3, transplanted with BALB/c CD45.2^+^ lineage-depleted hematopoietic stem and progenitor cells (HSPCs), and followed for up to 26 weeks (Figure 1, A and B). Four weeks after HCT, we observed donor engraftment in all peripheral blood lineages in mice conditioned with 75 cGy (Figure 1C). Stable mixed chimerism was maintained in blood throughout the 26-week experiment period in 5 of 5 mixed chimeric recipients (hereafter referred to as BALB/c:B6 mice; Figure 1D). Endpoint analysis of the spleen and bone marrow showed comparable chimerism levels (Figure 1, E and F). We also confirmed bone marrow engraftment of *donor* Lin^–^SCA1^+^cKIT^+^ (LSK) HSCs and persistence of *host* LSK HSCs (donor LSK chimerism: 34.2% ± 25.4%; Figure 1F). By contrast, we did not achieve durable multilineage mixed chimerism in B6 mice after conditioning with 50 or 25 cGy (Supplemental Figure 1, A–D; supplemental material available online with this article; https://doi.org/10.1172/jci.insight.194491DS1). Specifically, B6 mice conditioned with 50 or 25 cGy TBI did not generate donor-derived CD3^+^ T cells, which are crucial for allogeneic tolerance and stable mixed hematopoietic chimerism (33) (Supplemental Figure 1, A–D).

Using whole bone marrow (WBM) instead of lineage-depleted HSPCs achieved similar outcomes (Supplemental Figure 2). After HSPC or WBM transplantation, we did not observe signs of GVHD (e.g., mucosal changes, skin rash, or weight loss), and longitudinal body weight measures showed excellent weight gain from HCT to experimental endpoint in all groups (Supplemental Figure 1E and Supplemental Figure 2C). Thus, we established durable mixed hematopoietic chimerism in nondiabetic B6 mice across complete MHC mismatch with a NMA regimen combining 75 cGy TBI with αCD117, TCD, and baricitinib. These studies illustrate that systematic addition of reagents or steps could be useful for reducing TBI and conditioning toxicity, and we used this strategy to reduce TBI further. In all subsequent studies, we transplanted WBM to model the current clinical standard.

### Studies of bone marrow clearance after conditioning with αCD47 antibody and Bcl-2 inhibitor.

**Figure 2:**
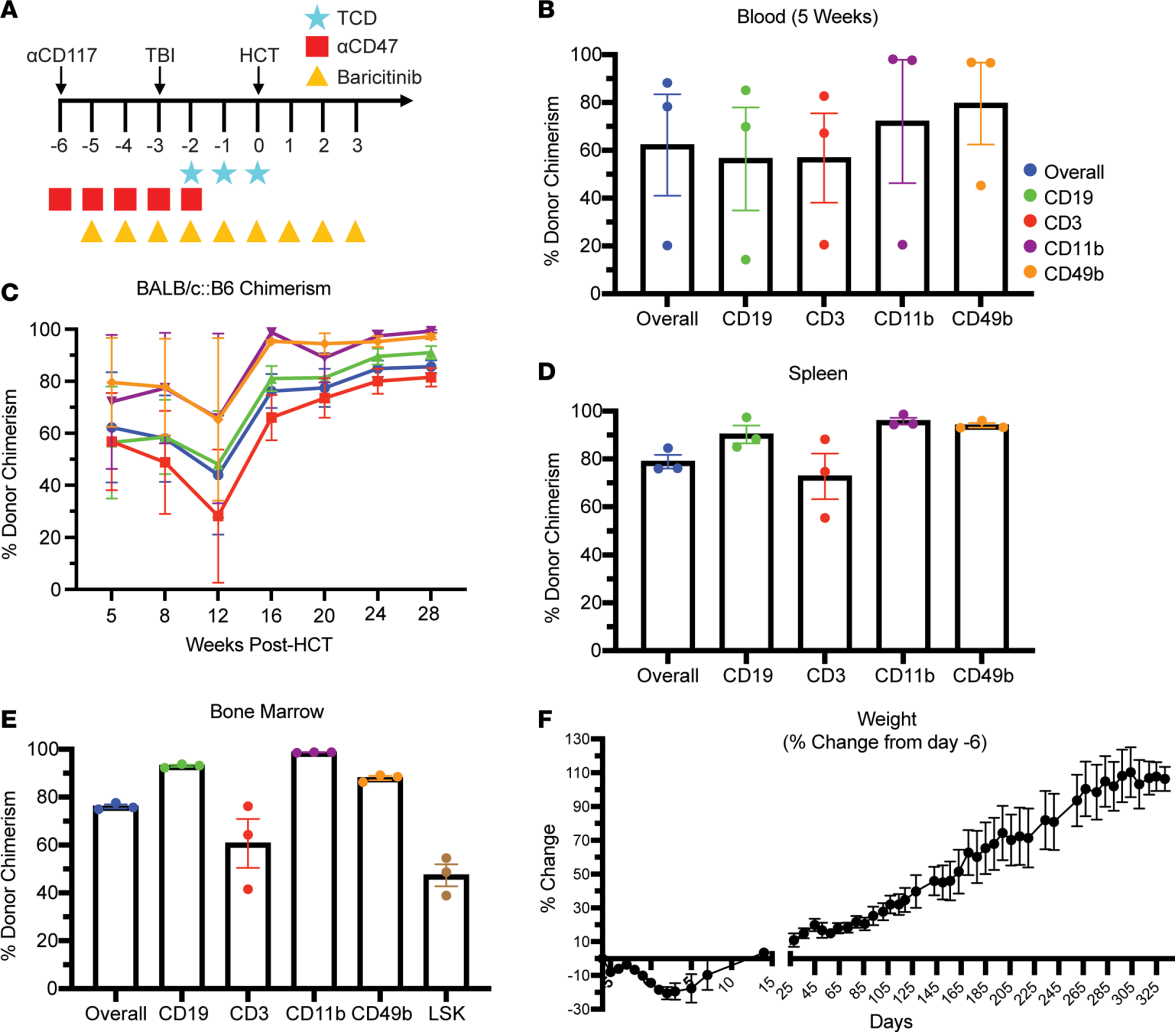
αCD47 monoclonal antibody conditioning promotes durable mixed hematopoietic chimerism with 25 cGy TBI. (**A**) Experimental conditioning and transplantation timeline. (**B**) Multilineage chimerism analysis of peripheral blood 5 weeks after HCT. (**C**) Longitudinal multilineage chimerism analysis of peripheral blood through 28 weeks after HCT. (**D**) Multilineage chimerism analysis of host spleen 47 weeks after HCT. (**E**) Multilineage chimerism analysis, including Lin^–^SCA1^+^cKIT^+^ (LSK) HSCs, of host bone marrow 47 weeks after HCT. (**F**) Weight of BALB/c:B6 mice overtime as a percentage of initial weight prior to conditioning start. *n* = 3 animals, from 1 experiment. Data are presented as mean ± SEM. TBI, total body irradiation; HCT, hematopoietic cell transplant; TCD, T cell depletion.

Prior studies have incorporated αCD47 antibodies into conditioning regimens to promote macrophage-mediated clearance of host HSCs and achieved mixed chimerism across a haplo-mismatched MHC barrier (30, 34). To evaluate conditioning with αCD47 to promote mixed chimerism across a full MHC mismatch, we conditioned B6 CD45.1^+^ mice with αCD117, baricitinib, TCD, and αCD47 antibody (300 g/d) from day –6 to day –2. On day –3 mice were irradiated with 25 cGy TBI and transplanted with 30E6 WBM cells from CD45.2^+^ BALB/c donors on day 0 (Figure 2A). Serial analysis of peripheral blood for up to 28 weeks after HCT revealed successful multilineage chimerism levels in B6 mice after conditioning with αCD47 antibody and 25 cGy TBI (Figure 2, B and C). Endpoint analysis of the spleen and bone marrow showed durable multilineage chimerism levels and engraftment of donor CD45.2^+^ LSK HSCs (donor LSK chimerism, 47.4% ± 7.9%; Figure 2, D and E). We did not observe indices of GVHD in BALB/c:B6 chimeras throughout our studies, including weight loss (Figure 2F), erythema or other skin changes. However, addition of conditioning αCD47 antibody resulted in approximately 20% reduction in body mass during the conditioning period, necessitating daily oral feeding supplementation for 7 days during and after conditioning (Figure 2F).

To reduce conditioning toxicity, we sought αCD47 dosage reductions. We measured bone marrow clearance after conditioning with αCD117, baricitinib, 30 cGy TBI, and either 75 or 50 g/d αCD47 (Supplemental Figure 3A). Conditioning with 75 and 50 g/d αCD47 antibody for 5 consecutive days reduced overall bone marrow cellularity and HSC count equally (Supplemental Figure 3, B and C). We observed little to no change in body mass of mice conditioned with 50 g/d, while the 75 g/d group experienced significant weight loss (Supplemental Figure 3D), a known complication of accompanying anemia (30, 34, 35). However, HSCs were not reduced to levels permissive to successful HCT engraftment with either αCD47 dose (Supplemental Figure 3C). These findings suggested that additional steps might be required with low-dose αCD47 to permit successful HCT engraftment and further reduction of conditioning TBI dosing.

Venetoclax is a pharmacologic inhibitor of Bcl-2, a survival factor in HSPCs and differentiated blood lineages, and was recently used to reduce conditioning TBI by 50% in nonhuman primates prior to successful mixed chimerism (25, 31). We evaluated the effect of venetoclax (200 g/d from days –6 to –2) on bone marrow clearance in mice conditioned with αCD117, baricitinib, and 50, 25, or 10 cGy TBI (Supplemental Figure 3E). Addition of venetoclax did not adequately clear HSCs, despite reduced overall cell counts (Supplemental Figure 3, F and G). However, venetoclax was well tolerated, causing only a mild, transient change in body mass that resolved by day 0 (Supplemental Figure 3H). We next investigated bone marrow clearance and mixed chimerism using αCD117-based conditioning with both venetoclax and αCD47.

### Durable allogeneic mixed hematopoietic chimerism after conditioning with 10 cGy TBI.

**Figure 3:**
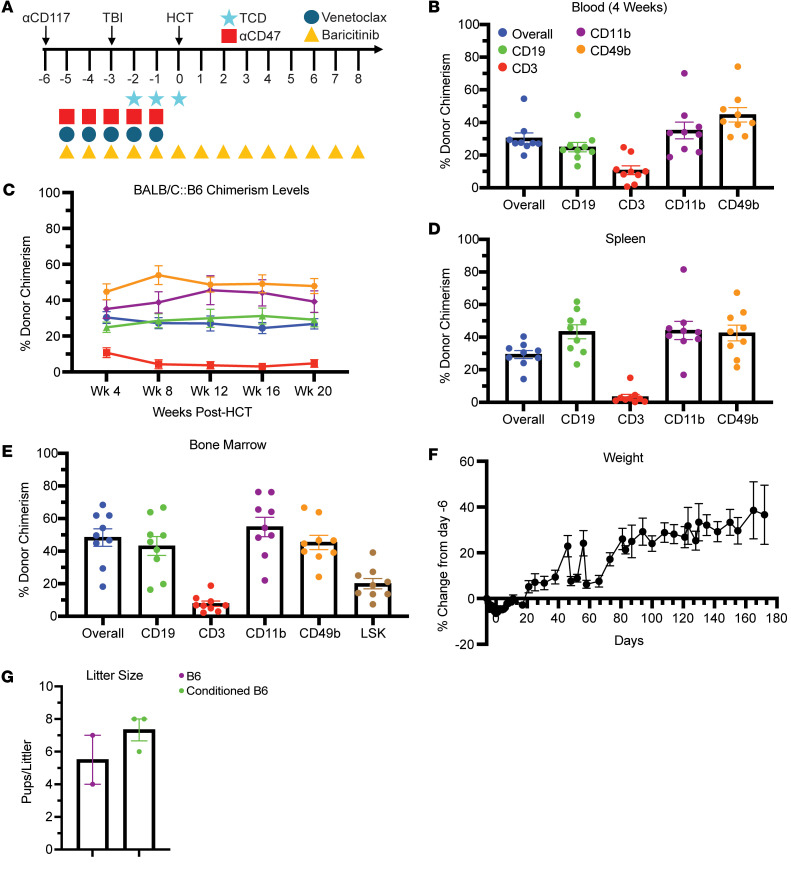
Durable mixed hematopoietic chimerism after nonmyeloablative conditioning with 10 cGy TBI. (**A**) Experimental conditioning and transplantation timeline. (**B**) Multilineage chimerism analysis of peripheral blood 4 weeks after HCT. (**C**) Longitudinal multilineage chimerism analysis of peripheral blood through 20 weeks after HCT. (**D**) Multilineage chimerism analysis of host spleen 22 or 25 weeks after HCT. (**E**) Multilineage chimerism analysis, including Lin^–^SCA1^+^cKIT^+^ (LSK) HSCs, of host bone marrow 22 or 25 weeks after HCT. (**F**) Weight of BALB/c:B6 mice overtime as a percentage of initial weight prior to conditioning start. (**B**–**F**) *n* = 9 animals from 2 independent experiments. (**G**) Litter sizes of unconditioned female mice paired with conditioned males (purple; *n* = 2 animals) and conditioned female mice paired with unconditioned males (green; *n* = 3 animals). Data are presented as mean ± SEM. TBI, total body irradiation; HCT, hematopoietic cell transplant; TCD, T cell depletion. See also, Supplemental Figure 4.

We conditioned B6 mice with venetoclax, αCD47 (50 g/d), αCD117 antibody, TCD, baricitinib, and 10 cGy TBI (Figure 3A). This conditioning regimen led to durable multilineage mixed hematopoietic chimerism after BALB/c WBM transplantation (Figure 3, B and C), with only modest, transient weight loss (<10%) (Figure 3F). Endpoint analysis of spleen and bone marrow 22 or 25 weeks after HCT revealed mixed chimerism and engraftment of donor CD45.2^+^ LSK HSCs in the bone marrow (Figure 3, D and E). By contrast, mice failed to achieve multilineage mixed chimerism and allogeneic tolerance after omission of αCD47 antibody or TBI (0 cGy) from this regimen (Supplemental Figure 4). Thus, systematic testing identified a combination of reagents that minimized conditioning XRT and permitted mixed chimerism in nondiabetic mice.

While this conditioning regimen was well tolerated, mice developed peritransplant anemia, corrected by a single whole blood transfusion. Despite the requirement for supportive intervention early on, conditioned mice showed normal average weight gain after conditioning alone or conditioning and HCT (Figure 3F). Importantly, both male and female mice remained fertile after conditioning (Figure 3G) and did not show indications of GVHD, including weight loss, erythema, or other skin changes.

### Diabetes reversal with allogeneic islet and bone marrow transplantation after NMA conditioning.

**Figure 4:**
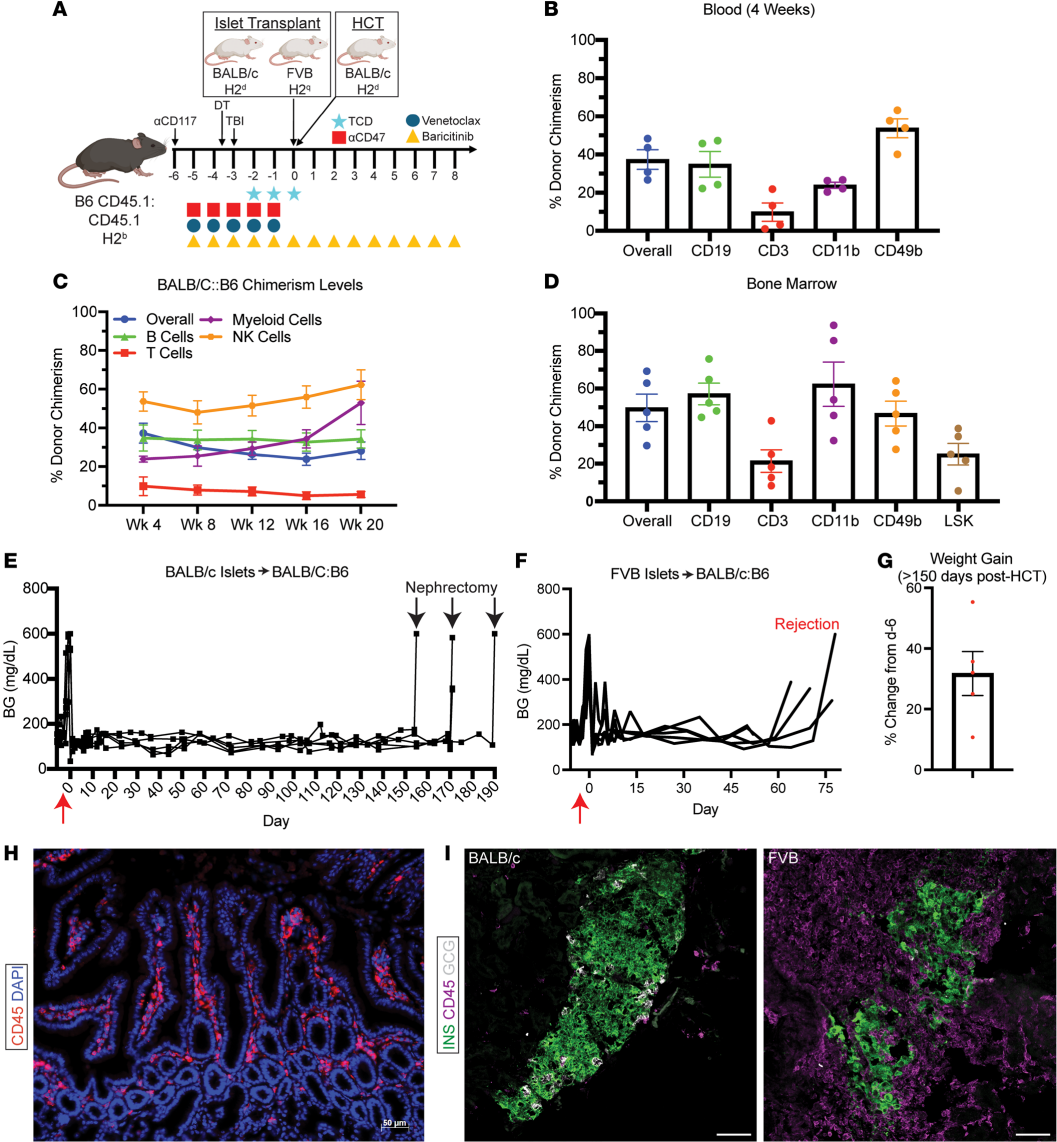
Allogeneic islet tolerance and diabetes reversal after simultaneous islet and bone marrow transplant in diabetic mice conditioned with 10 cGy TBI. (**A**) Experimental design and transplantation timeline. (**B**) Multilineage chimerism analysis of peripheral blood 4 weeks after HCT. (**C**) Longitudinal multilineage chimerism analysis of peripheral blood through 20 weeks after HCT. (**D**) Multilineage chimerism analysis, including Lin^–^SCA1^+^cKIT^+^ (LSK) HSCs, of host bone marrow 24 or 27 weeks after HCT. (**E**) Nonfasting blood glucose (BG) levels of diabetic B6 RIP-DTR mice that received simultaneous BALB/c bone marrow and islet transplants. (**B**–**E**) *n* = 5 animals from 3 independent experiments. (**F**) Nonfasting blood glucose levels of diabetic B6 RIP-DTR mice transplanted with BALB/c bone marrow and third-party FVB islets (*n* = 5 mice from 4 independent experiments). Red arrow indicates DT injection (**E** and **F**), and black arrows indicate nephrectomies (**E**). (**G**) Percentage weight change at day of nephrectomy compared with starting weight at day –6 (*n* = 5 animals). (**H**) Representative intestinal histology of BALB/c:B6 mice at 24 or 27 weeks after HCT, stained for CD45. *n* = 5 animals from 3 independent experiments. Scale bar: 50 μm. (**I**) Representative histology of BALB/c (left) and FVB (right) islets transplanted under the kidney capsule of BALB/c:B6 mice stained for insulin (green), CD45 (magenta), and glucagon (white). Scale bar: 50 μm. Data are presented as mean ± SEM. TBI, total body irradiation; HCT, hematopoietic cell transplant; TCD, T cell depletion.

We investigated if combined allogeneic HCT and islet transplantation could reverse established diabetes in B6 *RIP-DTR* mice after conditioning with αCD117, αCD47, TCD, venetoclax, baricitinib, and 10 cGy TBI (Figure 4A; see Methods, ref. 36). After injection of diphtheria toxin (DT; red arrow), all B6 *RIP-DTR* mice became severely diabetic, with glycemia >500 mg/dL (Figure 4, E and F). Prior to conditioning, diabetic mice were maintained on insulin (see Methods), which was discontinued on day 0 after HCT and islet transplantation. We transplanted BALB/c WBM and approximately 400 islets from BALB/c (donor-matched) or FVB (third-party) donors in the subcapsular renal space (Figure 4A). We observed multilineage mixed hematopoietic chimerism in peripheral blood of diabetic B6 *RIP-DTR* mice by 4 weeks after HCT (Figure 4B). Of 5 mice transplanted with BALB/c islets and followed for 20 weeks, all had durable chimerism (Figure 4C). Endpoint analysis of bone marrow revealed abundant donor chimerism, including LSK HSCs (Figure 4D). Thus, our NMA conditioning regimen incorporating only 10 cGy TBI achieved durable mixed hematopoietic chimerism and established allogeneic tolerance across full MHC mismatch in overtly diabetic mice.

Diabetic B6 *RIP-DTR* mice with durable chimerism (BALB/c:B6 *RIP-DTR*) maintained normal blood glucose levels throughout the follow-up period (>150 days) and did not require supplemental insulin or additional immunosuppression (*n* = 5/5; Figure 4E). We did not observe evidence of GVHD in chimeric BALB/c:B6 *RIP-DTR* mice; this included assessment of longitudinal body weight measures that showed normal average weight gain after HCT (Figure 4G) and gastrointestinal histology, which showed an absence of inflammation (Figure 4H).

Removal of the kidney harboring engrafted BALB/c islets (Nephrectomy; black arrows), resulted in rapid reversion to diabetes (*n* = 5/5; Figure 4E). Chimeric BALB/c:B6 *RIP-DTR* mice that received FVB islets had initial glycemic stabilization but spontaneously reverted to hyperglycemia by approximately 70 days after HCT, indicating rejection of these “third-party” islets and reestablishment of immune competence (*n* = 5/5; Figure 4F). Histology of the recovered islet graft at the experimental endpoint in BALB/c:B6 *RIP-DTR* mice revealed intact BALB/c islets with little to no CD45^+^ immune cell infiltrate. By contrast, FVB islet grafts showed heavy immune cell infiltration and reduced islet cells (Figure 4I). In summary, we have developed a conditioning regimen with 10 cGy TBI that promotes durable mixed hematopoietic chimerism, allogeneic graft tolerance, and diabetes reversal.

To determine whether this regimen induces durable tolerance to allogeneic antigens, nondiabetic B6 mice were conditioned and transplanted with BALB/c WBM and islets (Figure 4A). Then, 50 days after hematopoietic cell transplantation (HCT) and islet transplantation, chimeric mice received a second islet graft under the contralateral kidney capsule. Mice were euthanized 2 weeks later, and both grafts were evaluated histologically for evidence of rejection. No signs of acute allogeneic rejection or immune cell infiltration were observed in either the primary or secondary islet grafts (Supplemental Figure 5A). By contrast, we observed immune cell infiltration and reduction of “third-party” FVB islet cells (Supplemental Figure 5B).

To assess the presence of donor-reactive T cells, we isolated splenic T cells from CD45.2^+^ BALB/c:B6 chimeras, labeled these with CellTrace Far Red (Thermo Fisher Scientific), and then cultured these with CD11c^+^ splenic DCs from naive CD45.1^+^ B6, BALB c, or FVB mice. Both donor- (H2K^d+^) and host-derived (H2K^b+^) CD4^+^ and CD8^+^ T cells proliferated in response to third-party FVB DCs, whereas minimal proliferation was observed in response to either B6 or BALB/c DCs (Supplemental Figure 5, C–E, and Supplemental Figure 6). Together, these findings demonstrate that the regimen establishes stable donor-specific tolerance that supports acceptance of a secondary matched graft and is associated with deletion or functional control of donor-reactive T cells in BALB/c:B6 mixed chimeras.

### Central mechanisms of immune tolerance in mixed chimeric mice.

**Figure 5:**
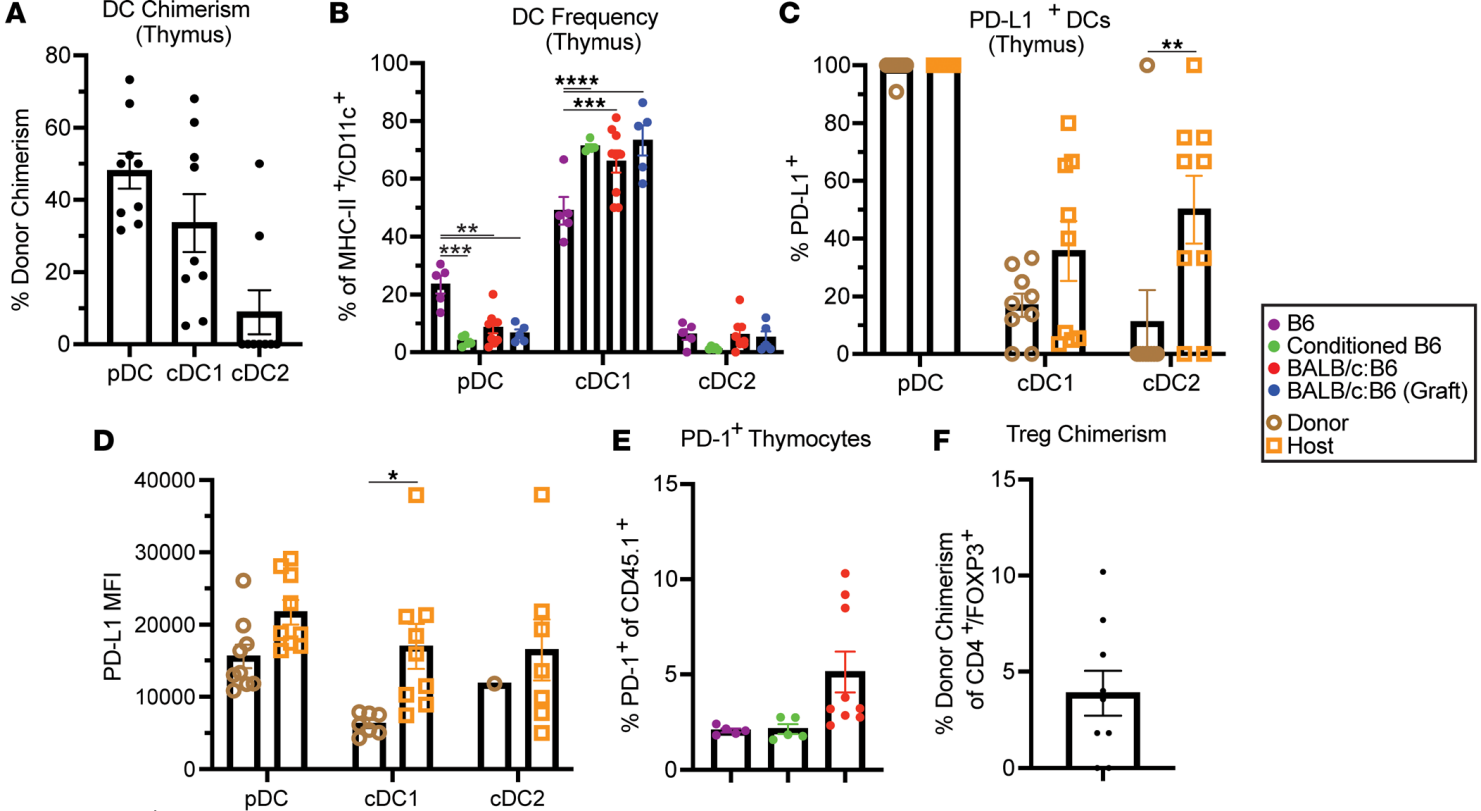
Central tolerance mechanisms in mixed chimeric mice. (**A**) Chimerism analysis of CD11c^+^MHC-II^+^ DC subsets in the thymus of B6:BALB/c chimeras 22 or 25 weeks after HCT. pDCs, B220^+^PDCA1^+^; cDC1, B220^–^SIRP^–^CD8^+^; cDC2, B220^–^SIRP^+^CD8^–^. (**B**) Thymic DC subset frequency in WT B6, conditioned B6, B6:BALB/c mixed chimeras, and B6:BALB/c mixed chimeras that received an islet graft. (**C**) Proportion and (**D**) MFI of PD-L1 expression in thymic host- and donor-derived DCs in B6:BALB/c mice. (**E**) Proportion of CD45.1^+^PD-1^+^ thymocytes in B6, conditioned B6, and B6:BALB/c mice. (**F**) Donor chimerism in CD4^+^FOXP3^+^ thymic Tregs. *n* = 5–9 animals from 2 independent experiments. “Conditioned B6” refers to B6 mice that received conditioning *without* HCT. Data are presented as mean ± SEM. (**B**–**D**) Two-way ANOVA with (**B**) Tukey’s or (**C** and **D**) Šidák’s post hoc test was used to determine significance. *P < 0.05, **P < 0.01, ***P < 0.001, ****P < 0.0001.

**Figure 6:**
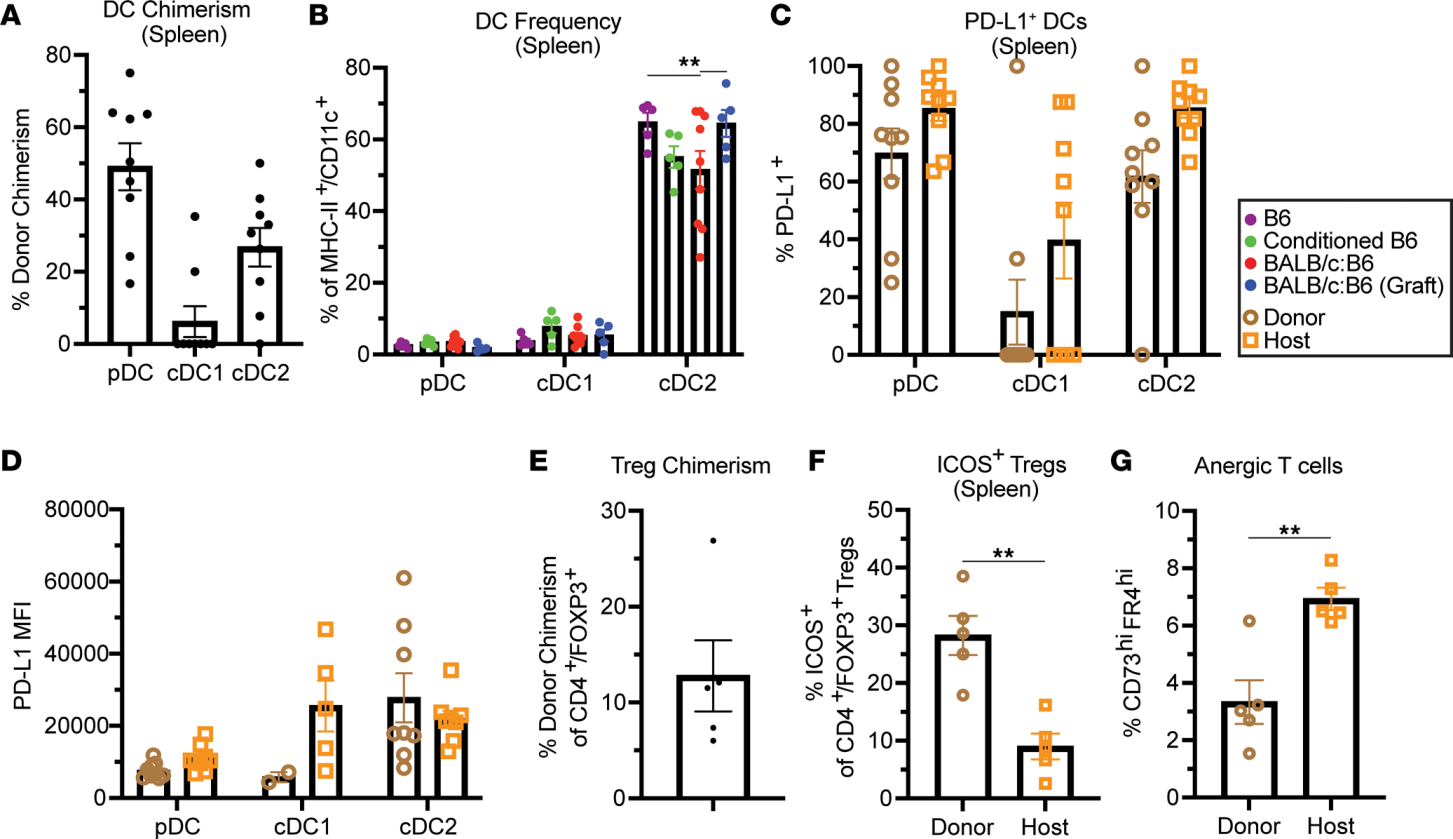
Peripheral tolerance mechanisms in mixed chimeric mice. (**A**) Chimerism analysis of CD11c^+^MHC-II^+^ DC subsets in the spleen of B6:BALB/c chimeras 22 or 25 weeks after HCT. pDCs, B220^+^PDCA1^+^; cDC1, B220^–^SIRP^–^CD8^+^; cDC2, B220^–^SIRP^+^CD8^–^. (**B**) Splenic DC subset frequency in WT B6, conditioned B6, B6:BALB/c mixed chimeras, and B6:BALB/c mixed chimeras that received an islet graft. (**C**) Proportion and (**D**) MFI of PD-L1 in splenic host and donor DCs in B6:BALB/c mice. (**E**) Donor chimerism in CD4^+^FOXP3^+^ splenic Tregs. (**F**) Proportion of ICOS^+^CD4^+^FOXP3^+^ splenic Tregs. (**G**) Frequency of CD73^hi^ FR4^hi^ anergic cells among host and donor CD4^+^ FOXP3^–^ conventional T cells (Tcon) cells of B6:BALB/c spleen. *n* = 5–9 animals from 2 independent experiments. “Conditioned B6” indicates B6 mice that received conditioning *without* HCT. Data are presented as mean ± SEM. (**B**) Two-way ANOVA with Tukey’s post hoc test or unpaired *t* test (**F** and **G**) were used to determine significance. **P < 0.01.

Thymic DCs augment “negative selection” of T cells in the thymus (37–40). In mixed chimerism, donor antigen-presenting cells in thymus, including thymic DCs, present both donor and self-antigens to developing thymocytes, promoting autologous and allogeneic tolerance (41). To assess tolerance mechanisms in BALB/c:B6 chimeras, we characterized the major CD11c^+^ DC subsets: B220^+^PDCA1^+^ plasmacytoid DCs (pDCs), conventional CD8^+^SIRP^–^ cDC1 cells, and CD8^–^SIRP^+^ cDC2 cells in the thymus and spleen of chimeric BALB/c:B6 mice compared with controls (37–40, 42). We confirmed the presence of CD45.2^+^ donor-derived DCs in both the thymus (Figure 5A and Supplemental Figure 7A) and spleen (Figure 6A and Supplemental Figure 7F) of mixed chimeric mice, indicative of antigen presentation by donor DCs in these tissues. Compared with both unconditioned and conditioning-only control B6 mice, thymic cDC2 DCs were detected at similar frequencies in BALB/c:B6 mice (Figure 5B and Supplemental Figure 7B). Interestingly, the frequency of pDCs was significantly reduced in conditioned controls and BALB/c:B6 mice, while cDC1s were significantly increased all other groups, compared with naive B6 mice (Figure 5B and Supplemental Figure 7B). These differences in DC frequencies highlight potential shifts in immune regulation in mixed chimerism, warranting further exploration of pathways like the PD-1/PD-L1 signaling axis, which is critical for maintaining central and peripheral tolerance (43, 44).

In the thymus, PD-1 is upregulated in thymocytes undergoing negative selection and primarily localized to the medulla, while PD-L1 is expressed in both the cortex and medulla by thymic epithelial and DCs (44–46). We observed an increase in the frequency and levels of PD-L1 expression in host-derived cDC1 and cDC2 DCs of mixed chimeric mice (Figure 5, C and D, and Supplemental Figure 7C). We also observed an increase in the overall frequency of PD-1^+^ thymocytes in chimeric mice, compared with controls (Figure 5E and Supplemental Figure 7D). To further evaluate the selection of donor and host-derived thymocytes, we analyzed T cells with the V11 domain, which are deleted by genome-encoded superantigen presentation in BALB/c but not B6 mice (47). In BALB/c:B6 mice, approximately 70% of Vβ11^+^ T cells were depleted, compared with B6 controls (Supplemental Figure 8A). Importantly, we observed deletion of both donor (CD45.1^+^) and host (CD45.2^+^) Vβ11^+^ T cells, indicating education of host T cells to donor antigens (Supplemental Figure 8B). By contrast, Vβ8.1^+^ T cells, which are not under selective pressure in BALB/c or B6 strains, were not depleted (Supplemental Figure 8C). Negative selection of thymocytes is often accompanied by the presence of thymic regulatory T cells (tTregs), and we detected donor-derived CD4^+^/FOXP3^+^ tTregs in the thymus of chimeric BALB/c:B6 mice (Figure 5F and Supplemental Figure 7E). Together, these results support mechanisms of central tolerance induction in BALB/c:B6 mixed chimeras, where donor DCs in the host thymus could mediate negative selection and augment the generation of host and donor-derived tTregs.

### Peripheral mechanisms of immune tolerance in mixed chimeric mice.

Peripheral tolerance mechanisms are crucial for suppressing allo- and autoreactive T cells that evade thymic selection and for tolerizing residual tissue-resident host T cells that survive conditioning. As with thymic DCs, we evaluated both the frequency of splenic DC subsets and their PD-L1 expression (Figure 6, B–D, and Supplemental Figure 7, G and H). Similarly, we observed an upward trend in the frequency and level of PD-L1 expression in host-derived splenic DCs (Figure 6, C and D). These findings indicate a role for PD-L1 expression in splenic DCs in promoting peripheral tolerance.

Peripheral tolerance in transplantation is also associated with Treg activity (48). Consistent with our observations in the thymus, we observed donor-derived splenic CD4^+^FOXP3^+^ Tregs in mixed chimeric mice (Figure 6E and Supplemental Figure 7I). To determine whether Tregs present in mixed chimeric mice were functional, we analyzed inducible costimulator (ICOS), whose expression is correlated with increased IL-10 secretion and suppression potential, in donor- and host-derived splenic Tregs (49). While both host- and donor-derived ICOS^+^ Tregs are present, the frequency of donor-derived ICOS^+^ Tregs was significantly higher than host-derived ICOS^+^ Tregs (Figure 6F and Supplemental Figure 7J). Donor-derived Tregs can suppress alloreactive host-type T cells (50). Supporting this possibility, we observed that the proportion of host-derived anergic CD4^+^CD73^hi^FR4^hi^ cells was significantly higher (Figure 6G and Supplemental Figure 7K). Additionally, myeloid-derived suppressor cells (MDSCs) have been shown to play a role in promoting tolerance to allogeneic tissues (51, 52). In mice, MDSCs are broadly categorized as granulocytic (G) (CD11b^+^, LY6G^+^, LY6C^lo^) or monocytic (M) (CD11b^+^, LY6G^–^, LY6C^hi^) MDSCs, which differ in lineage and suppressive mechanisms (53). Thus, we evaluated MDSCs in the peripheral blood and spleens of mixed chimeric mice and observed G- and M-MDSCs at comparable proportions as in control B6 mice, with both populations exhibiting donor chimerism (Supplemental Figure 8, D–G). In summary, durable mixed hematopoietic chimerism after conditioning with our low-dose radiation regimen likely reflects both central and peripheral mechanisms that establish and maintain tolerance to donor HSCs and islets and prevent GVHD.

## Discussion

Mixed hematopoietic chimerism can induce allogeneic, donor-specific tolerance across MHC-mismatched barriers. Indeed, in patients suffering renal failure who received donor-matched HCT and kidney allotransplants, this approach has achieved decades-long renal replacement without systemic immunosuppression (7, 9, 54, 55). Despite demonstrated proof of concept in humans, broader use of mixed hematopoietic chimerism for tolerance induction is limited by the toxicities associated with conventional HCT transplant protocols that use high doses of radiation or DNA-damaging chemotherapy (41, 56). Pretransplant conditioning is necessary to create niche space in the host bone marrow donor HSCs to engraft and to prevent immune rejection of transplanted cells (13). Thus, we developed a NMA condition regimen that supports durable multilineage mixed hematopoietic chimerism after nearly complete elimination of conditioning irradiation. Transplantation of donor-matched islets in diabetic, immune competent mice, resulted in durable diabetes reversal without chronic immunosuppression or complications like GVHD. This represents a substantial advance from our prior work (22).

Chemotherapeutics and radiation doses currently used for HCT conditioning are associated with myriad morbidities, including impaired endocrine function, fertility loss, and increased risk for secondary malignancy (57). Here, we demonstrate that the nongenotoxic conditioning agents incorporated in our conditioning regimen are well tolerated, do not affect pancreatic islet function, and maintain other measures of functional status, like fertility. A single dose of TBI <10 cGy is operationally defined as “low dose” and is not associated with increased carcinogenic risk (58); thus, we sought to reduce conditioning TBI to ≤10 cGy, with the aspiration to broaden adoption of mixed chimerism for organ replacement. To achieve donor cell engraftment with reduced irradiation, we systematically assessed conditioning reagents currently in clinical use or involved in clinical trials. Baricitinib (59), venetoclax (60), CD4 monoclonal antibody (ibalizumab) (61), and NMA radiation are all currently approved for the treatment of multiple nonmalignant or malignant disease. Human CD117 Ab (JSP191) is in multicenter trials (ClinicalTrials.gov, NCT02963064, NCT04429191, NCT04784052) and has shown a good safety profile and promising results. Likewise, numerous CD47 monoclonal antibodies are currently under clinical evaluation (62). While the development and clinical evaluation of CD8 monoclonal antibodies is limited, transient TCD can also be achieved with antithymocyte globulin or other conditioning agents currently in clinical use.

The observed variability in total and T cell chimerism across conditioning regimens likely reflect differences in both the intensity of host immune depletion and support for donor hematopoietic engraftment. First, conditioning intensity alters bone marrow niche availability and host myelosuppression, which directly affects donor cell engraftment and subsequent multilineage reconstitution (5, 63, 64). Second, differences in the composition and dose of the graft and the degree of peritransplant TCD or costimulation blockade can affect the kinetics and magnitude of donor cell reconstitution in the periphery (5, 65). Third, residual host immunity and homeostatic proliferation can favor expansion of host over donor T cells or selectively permit donor myeloid engraftment while limiting donor lymphoid reconstitution (66).

GVHD is a potentially life-threatening complication of HCT and an impediment to expanding HCT for nonmalignant disorders (67). Conditioning intensity and HSC sourcing and composition are major components of GVHD risk (68–70). While enriched HSPC populations, depleted of mature effector cells, can be used to mitigate GVHD risks associated with WBM preparations, enriched HSPCs do not engraft as well as WBM (71, 72). We achieved similar chimerism outcomes with our conditioning regimen and either WBM or lineage-depleted HSPC transplantation and observed no signs of GVHD, like hair loss, rashes, postural changes, or weight loss, following either. On the contrary, longitudinal body weight measures showed excellent weight gain from HCT to experimental endpoint in all comparison groups. Baricitinib use to prevent and treat GVHD has been evaluated in animal models and is currently being evaluated for clinical treatment of GVHD (73, 74). Thus, in addition to being a key element of our NMA conditioning regimen, baricitinib might both suppress peritransplant acute GVHD and prevent chronic GVHD.

To assess mechanisms of allotolerance in B6 mice with mixed chimerism, we characterized cellular and molecular features of central and peripheral tolerance. We observed durable mixed chimerism in all immune cell lineages and compartments evaluated, including the bone marrow, spleen, thymus and circulating blood. Furthermore, the data support a role for thymic-mediated central tolerance, including evidence of donor antigen-presenting cell chimerism and elimination of donor-reactive Vβ11^+^ T cells, in promoting allogeneic tolerance in these settings. Lack of islet immune cell infiltration in mixed chimeric B6 *RIP-DTR* mice after HCT and islet transplantation suggests that donor alloantigen tolerance was achieved using mixed chimerism. Maintenance of immune competence after NMA conditioning and islet transplantation to achieve diabetes reversal is an important goal for clinical translation. We observed robust rejection of third-party FVB islet grafts by mixed chimeric BALB/c:B6 mice, indicating reconstituted immune function. To further evaluate the durability of tolerance to allogeneic antigens, BALB/c:B6 chimeric mice were challenged with a second islet graft transplanted at least 50 days after the initial islet graft and HCT. Two weeks following the second transplant, neither the primary nor secondary graft showed evidence of acute or chronic rejection. These findings were supported by MLR analysis of splenic T cells from BALB/c:B6 chimeras cultured with B6, BALB/c, or FVB DCs. BALB/c:B6 T cells exhibited robust proliferation in response to third-party FVB DCs, while demonstrating minimal proliferation when stimulated with B6 or BALB/c DCs. Together, these results indicate that the regimen induces stable donor-specific tolerance capable of supporting long-term acceptance of a secondary matched graft and is associated with deletion or functional restraint of donor-reactive T cells in BALB/c:B6 mixed chimeras. Future mechanistic studies could include evaluation of additional mixed chimerism features, like medullary thymic epithelial cells.

To advance this approach toward clinical implementation, several key steps remain. First, further refinement of reduced-intensity conditioning regimens will be critical to minimize toxicity while preserving engraftment and tolerance. Second, validation in other stringent and clinically relevant models is needed to ensure robustness across diverse immune settings and disease contexts, including with NOD mice (75). Finally, careful evaluation of the safety and durability of tolerance will be essential, including monitoring for potential off-target effects and long-term stability of immune regulation. Together, these considerations highlight the path forward for translating this strategy into the clinic and underscore the challenges that must be addressed to realize its therapeutic potential.

Building on this framework, we demonstrate the use of a clinically portable NMA conditioning regimen combined with islet transplantation to establish mixed hematopoietic chimerism and allogeneic tolerance across full MHC barriers. Features of this strategy, including a unique combination of conditioning agents with synergistic mechanisms that minimize irradiation, represent an exciting conceptual advance for islet allotolerance and may facilitate clinical adoption in diabetes. Importantly, these findings could be extended to promote tolerance of other solid organ transplants, potentially eliminating the need for chronic immunosuppression. Future work will further explore whether this strategy can be adapted to reverse autoimmune diabetes and other autoimmune disorders and to induce tolerance to replacement islet cells derived from renewable sources, including multipotent stem cells.

## Methods

### Sex as a biological variable.

Our study examined male and female animals, and similar findings are reported for both sexes.

### Animals.

Female and male B6 CD45.1 (stock no. 002014), BALB/c (stock no. 000651), and FVB (stock no. 001800) mice were purchased from The Jackson Laboratory. B6 *RIP-DTR* mice were generated and maintained by our group and used at 10–20 weeks of age (36). This strain expresses the *Ins2-HBEGF* (RIP-DTR) transgene and the mutant *Ptprca* (CD45.1) allele on the B6 mouse background. The *RIP-DTR* transgene allows for rapid induction of diabetes by β cell–specific ablation and 100% penetrance with a single i.p. injection of DT in males and females. Healthy euglycemic littermates of the same sex were randomly assigned to experimental groups and were not involved in any prior procedures. For MLR assays, WT B6 (stock no. 000664) and CByJ.SJL(B6)-Ptprca/J (BALB-CD45.1; stock no. 006584) mice were purchased from The Jackson laboratory and utilized as HCT recipients or DC donors. All animals were fed standard chow and water ad libitum and housed in non-specific-pathogen-free conditions at the Stanford School of Medicine. Animal experiments were approved by the Stanford Administrative Panel on Laboratory Animal Care (IACUC).

### Diabetes induction, monitoring, and maintenance.

A 1-time (25 ng/g body weight) injection of DT (Cayman Chemical) in normal saline was administered i.p. in adult mice to induce diabetes from cell ablation. Their body weight and blood glucose were recorded daily using True Metrix Blood Glucose Monitor and Test Strips (Trividia Health). Diabetic mice were defined as have nonfasting blood glucose >250 mg/dL for 2 or more days in a row. To stabilize blood glucose on the morning of islet transplant, mice received a 1-time dose of 40 U/kg insulin glargine (Sanofi) in normal saline. After islet transplant, mice were monitored daily for wellness for up to 10 days, after which body weight and blood glucose levels were recorded once per week. Mice are considered euglycemic when their nonfasting blood glucose returns to <250 mg/dL.

### Conditioning, reagents, and equipment.

A graphical timeline of conditioning is shown in Figure 3A (see Supplemental Table 1). Mice were given 500 μg diphenhydramine HCl i.p. approximately 10–15 minutes prior to αCD117. 500 μg αCD117 was injected retro-orbitally into mice under isoflurane anesthesia on day –6 prior to HCT. Mice were irradiated on day –3 TBI. 300 μg each of αCD4 and αCD8 was administered i.p. on days –2, –1, and 0. The selective JAK1/2 inhibitor, baricitinib, was dissolved in 100% DMSO at 20 mg/mL and stored at –20°C in 100 μL aliquots. DMSO stocks were thawed and diluted 1:10 in PBS immediately prior to use, and 200 μL/mouse was injected s.c. (400 μg). Baricitinib was dosed days –5 to +3 or +8, according to timelines. αCD47 was administered on days –6 to –2 (Figure 2 and Supplemental Figure 3) or days –5 to –1 (Figures 3 and 4 and Supplemental Figure 4) at the stated concentrations. The selective Bcl-2 inhibitor, venetoclax, was dissolved in 100% DMSO at 20 mg/mL and stored at –20°C in 50 μL aliquots. Immediately prior to use, DMSO stocks were thawed and diluted in 400 μL PEG-300, 50 μL Tween 80, and 500 μL saline; solvents were added in this order and mixed thoroughly in between. 200 μL/mouse (200 μg) venetoclax was injected i.p. Venetoclax was administered on days –5 to –1. Animal irradiation (XRT) was performed in a Kimtron Polaris IC-250 Biological Irradiator with a 225 kV X-ray tube filtered by 0.5 mm Cu source set at 225 kV, 13.3 mA. Mice were divided in irradiation pie cages from Braintree Scientific when irradiated. Dosimetry calibration for our setup was performed using published methods on radiochromic film dosimetry (76).

### Bone marrow isolation, enrichment, and transplant.

Donor BALB/c mice (6–7 weeks old) were euthanized, and femurs, tibias, and vertebral bodies were collected. Bones were crushed via mortar and pestle in PBS with 2% FBS, 10 mM HEPES, and 2 mM EDTA to recover WBM. WBM was filtered through a 70 μm cell strainer, and RBCs were lysed in RBC Lysis Buffer (BioLegend). WBM cells were stained with Trypan Blue (Stemcell Technologies) and counted with a Countess 3 Automated cell counter (Thermo Fisher Scientific). Lineage-negative (Lin^–^) bone marrow cells were enriched by magnetic column separation using a Lineage Cell Depletion cocktail (Miltenyi Biotec) as per manufacturer’s instructions. 1.5E6 Lin^–^ cKIT^+^ cells or 30E6 WBM cells resuspended in 100 μL PBS were injected retro-orbitally. Lin^–^cKIT^+^ HSPC preparation composition has been previously described (22).

### Islet isolation and transplantation.

Islet isolation and transplantation was performed as previously described with minor modifications (77, 78). Briefly, pancreases were perfused with 100–125 μg/mL Liberase TL (Roche Diagnostics) through the common bile duct and digested in a 37°C water bath for 18–22 minutes. After washing with Hank’s Buffered Saline (HBS; Caisson Labs), the crude digest was purified over a discontinuous density gradient, washed once more with HBS, and cultured overnight in 5.5 mM glucose RPMI 1640 (Corning) supplemented with 10% FBS, 10 mM HEPES, and 1% penicillin-streptomycin solution. Recipient mice were anesthetized with a ketamine/xylazine mix and given s.c. analgesics. 200–400 islets were injected under the kidney capsule of recipient mice with a microcapillary, as described previously (79). The nephrectomy procedure involved the same anesthetic regimen as islet transplantation, and renal vessels were first tied to prevent hemorrhage before the kidney containing islet graft was removed.

### Histology.

Islet graft-bearing kidneys, pancreases, and intestines were fixed in 4% paraformaldehyde overnight, incubated overnight in 30% sucrose, embedded in optimal cutting temperature compound, and frozen on dry ice. 6–10 μm sections were made on a Leica CM3050 S (Leica Biosystems). Immunofluorescent staining was performed using standard methods. Briefly, sections were blocked for 1 hour and then incubated with primary antibodies overnight at 4°C. Sections were washed for 5 minutes 3 times before incubation with secondary antibodies for 2 hours at room temperature or overnight at 4°C and washed 3 times for 5 minutes again. Slide covers were set with Hard-set Mounting Medium with DAPI (Santa Cruz Biotechnology). Slides were imaged on Zeiss AxioM1 or Leica SP2 confocal microscopes. Postprocessing and color channel merging was performed in Fiji (80). Primary and secondary antibodies and dilutions are documented (Supplemental Table 2).

### Peripheral blood, spleen, thymus, and BM preparation for flow cytometry.

100 mL of whole blood was collected via the tail vain into EDTA-coated tubes. Spleens and thymuses were directly mashed through a 70 mm cell strainer. Bone marrow cells were isolated as above. Samples underwent RBC lysis in RBC Lysis Buffer (BioLegend) for 5–10 minutes at 4°C before downstream staining for analysis.

### Flow cytometry analysis.

Gating strategies were previously described (22) (Supplemental Figure 9). For analysis of mixed chimerism, cells were first stained with LIVE/DEAD Fixable Near-IR Dead Cell Stain Kit (Thermo Fisher Scientific) and blocked with TruStain FcX anti-mouse (BioLegend) for 10 minutes on ice in Cell Stain Buffer (BioLegend). Antibodies used for staining were from BioLegend. Extracellular markers were stained with antibodies listed (Supplemental Table 2) at manufacturer’s recommended dilutions. Staining of intracellular markers was conducted with BioLegend True-Nuclear Transcription Factor Buffer Set as per manufacturer’s instructions. Cells were analyzed with a 5L Aurora (Cytek Biosciences). Data were analyzed using FlowJo (10.9).

### Mixed lymphocyte reaction.

Details of the mixed lymphocyte reaction assay are provided in the Supplemental Methods.

### Statistics.

Statistical details of all experiments can be found in the figure legends and Results, including the value of *n*. All data are represented as mean ± SEM, where *n* represents number of animals. Animals were randomly assigned to experimental groups, and all samples represent biological replicates. Statistical analysis was performed using Prism 10 (GraphPad). Differences between the means of two groups were tested using unpaired 2-tailed Student’s *t* test with Welch’s correction. Differences between the means of 3 or more groups were tested by 2-way ANOVA using Tukey’s multiple comparisons test with Geisser-Greenhouse correction. Some data were excluded by Prism 10’s outlier function. Sample size estimates were not used. A *P* value of 0.05 or less was considered statistically significant.

### Study approval.

Animal experiments were approved by the Stanford Administrative Panel on Laboratory Animal Care (IACUC) in line with ARRIVE guidelines.

### Data availability.

All data generated or analyzed during this study are included in this published article (and its supplemental materials) or are available from the corresponding author on reasonable request. The Supporting Data Values file includes values underlying graphed data and reported means presented in both the main text and supplemental materials.

## Author contributions

SAR designed and performed experiments, data collection, and data analysis; wrote the manuscript; and was involved in funding acquisition. PB advised on experimental design and assisted in performing experiments, data collection, data analysis, and visualization; edited the manuscript; and was involved in funding acquisition. DMB performed experiments, data collection, data analysis, and visualization. XG, RR, NN, and MN performed experiments and data collection. JAS and SP provided guidance and feedback on experimental design and results, provided reagents, reviewed and edited the manuscript, and were involved in funding acquisition. SKK designed experiments, wrote the manuscript, supervised the project, acquired funding, and is the guarantor of this work.

## Conflict of interest

SAR is a consultant and stockholder of Tolerance Bio Inc. JAS is a cofounder, stockholder, and board member of Jasper Therapeutics Inc.

## Funding support

This work is the result of NIH funding, in whole or in part, and is subject to the NIH Public Access Policy. Through acceptance of this federal funding, the NIH has been given a right to make the work publicly available in PubMed Central.

- NIH grants R01 DK107507, R01 DK108817, U01 DK123743, and P30 DK116074 to SKK, T32 GM736543 to PB, and 1TL1DK139565-01 and F32DK141209 to SAR.
- Stanford Interdisciplinary Graduate Fellowship through Bio-X to PB.
- Stanford Institute for Immunity, Transplantation & Infection and Stanford Autoimmune & Allergy Supergroup 281539 to SKK and SAR.
- Breakthrough T1D Northern California Center of Excellence 5-COE-2019-860-S-B to SKK and JAS.
- Stanford VPUE Research Fellowship to DMB.
- Breakthrough T1D Postdoctoral Fellowship 3-PDF-2024-1502-A-N to MN.

## Supplementary Material

Supplemental data

Supporting data values

## Version 1 04/21/2026

In-Press Preview

## Version 2 06/08/2026

Electronic publication
