# Supplementary material for: Improved conditioning for hematopoietic chimerism induces islet tolerance to cure diabetes
Source: JCI Insight. 2026 Apr 9;11(11):e194491. doi: 10.1172/jci.insight.194491 (PMC13313546; doi:10.1172/jci.insight.194491)
Supplement: Supplemental data [file jciinsight-11-194491-s059.pdf]

Improved conditioning for hematopoietic chimerism induces islet tolerance to cure diabetes

Stephan A. Ramos[1], Preksha Bhagchandani[1], Diego M. Burgos[1], Xueying Gu[1], Richard Rodriguez[1], Nadia Nourin[1], Martin Neukam[1], Shiva Pathak[2], Judith A. Shizuru[2,3,4], Seung K. Kim[1,3,4,*]

[1] Department of Developmental Biology, Stanford School of Medicine; Stanford, CA 94305, USA

[2] Division of Blood and Marrow Transplantation, Department of Medicine, Stanford School of Medicine; Stanford, CA 94305, USA

[3] Stanford Diabetes Research Center, Stanford School of Medicine; Stanford, CA 94305, USA

[4] Northern California Breakthrough T1D Center of Excellence, Stanford School of Medicine; Stanford, CA 94305, USA

*Corresponding author. Email: seungkim@stanford.edu

Conflict of Interest

S.A.R is a consultant and stockholder of Tolerance Bio, Inc. J.A.S. is a co-founder, stockholder, and board member of Jasper Therapeutics, Inc.

Supplementary Materials

Supplementary Experimental Procedures

Preparation of T cells for MLR Assay

In vitro mixed lymphocyte reaction assays were performed as described previously (1). In brief, spleens harvested from donor mice (BALB/c:B6, WT B6, or WT BALB/c) were mashed and strained through 70 µm filters. After RBC lysis (BioLegend: cat # 420302), pan T cells were magnetically enriched using the EasySep Mouse T cell Isolation Kit (StemCell Technologies: Cat # 19851). Enriched T cells were labeled with CellTrace Far Red (Thermo Fisher Scientific: cat # C34564), per the manufacturer's instructions. Enriched T cells were resuspended in complete RPMI media and added to round-bottom 96-well plates at 2 x 10^5 cells per well.

Preparation of APCs cells for MLR Assay

Spleens were harvested from donor mice (B6-CD45.1, BALB/c-CD45.1, and FVB), processed, and enriched for $CD11c^+$ dendritic cells (DCs) using the EasySep Mouse CD11c Positive Selection Kit II (Stemcell Technologies: cat # 18781), following the manufacturer's instructions. Enriched DCs were resuspended in complete RPMI media and added wells at 2 x 10^4 cells per well.

In vitro mixed lymphocyte reaction

Enriched T cells and DCs were added to round-bottom 96-well plates in complete RPMI media. Complete RPMI media consisted of 10% heat inactivated FBS, 1x GlutaMax (Thermo Fisher Scientific: cat # 35050061), 1x Pen/Strep (Thermo Fisher Scientific: cat # 15140122), 10mM HEPES (Thermo Fisher Scientific: cat# 15630080), 1x NEAA (Thermo Fisher Scientific: cat # 11140050), 1mM Sodium Pyruvate (Thermo Fisher Scientific: cat # 11360070), and 50 µM β-mercaptoethanol (Thermo Fisher Scientific: cat # 31350010). After 3 days of culture, cells were stained with antibodies against CD3, CD4, CD8, CD45.1, CD45.2, $H2K^b$, and $H2K^d$ and loss of CellTrace Far Red in donor- and host-derived $CD4^+$ and CD8 T^+ cells was assessed by flow cytometry.

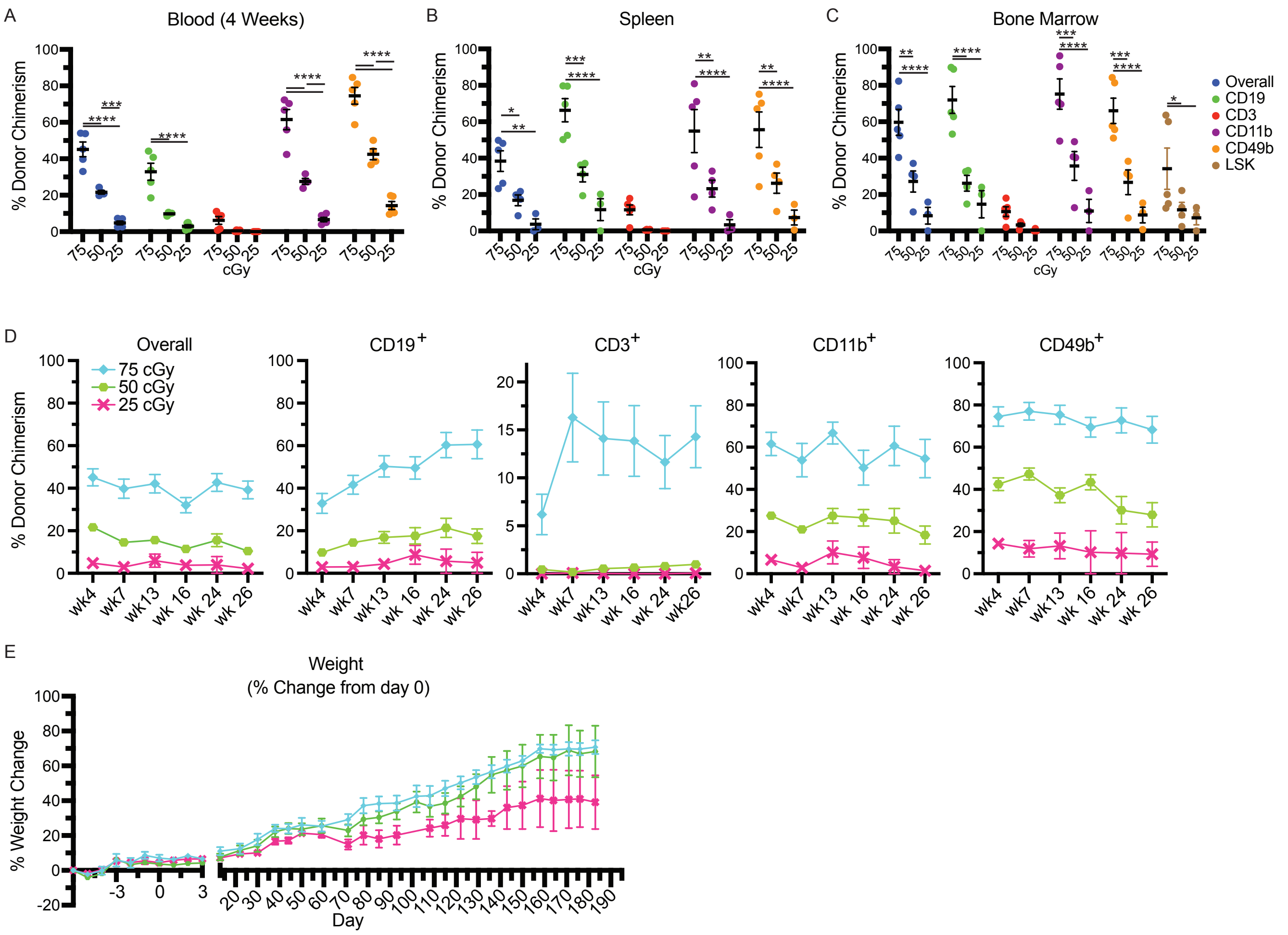

Supplementary Figure S1: Lack of durable mixed hematopoietic chimerism after conditioning with baricitinib and ≤50 cGy TBI. (A) Multilineage chimerism analysis of peripheral blood 4 weeks post-HCT in non-diabetic B6 mice conditioned as outlined in Fig 1B with 75, 50, or 25 cGy TBI (n = 5, 4, and 5 for 75, 50, and 25 cGy groups, respectively). **(B)** Multilineage chimerism analysis of host spleen 26 weeks post-HCT. **(C)** Multilineage chimerism analysis, including Lin$^-$Sca1$^+$cKit$^+$ (LSK) HSCs, of host bone marrow 26 weeks post-HCT. (A-C) Data were analyzed by two-way ANOVA with Tukey's post hoc test. **(D)** Longitudinal multilineage chimerism analysis of peripheral blood through 26 weeks post-HCT. **(E)** Weight after conditioning and HCT as a percentage of starting weight. (C-E) n = 5, 4, and 3 animals for 75, 50, and 25 cGy groups, respectively, from one experiment. Data presented as mean ± SEM.

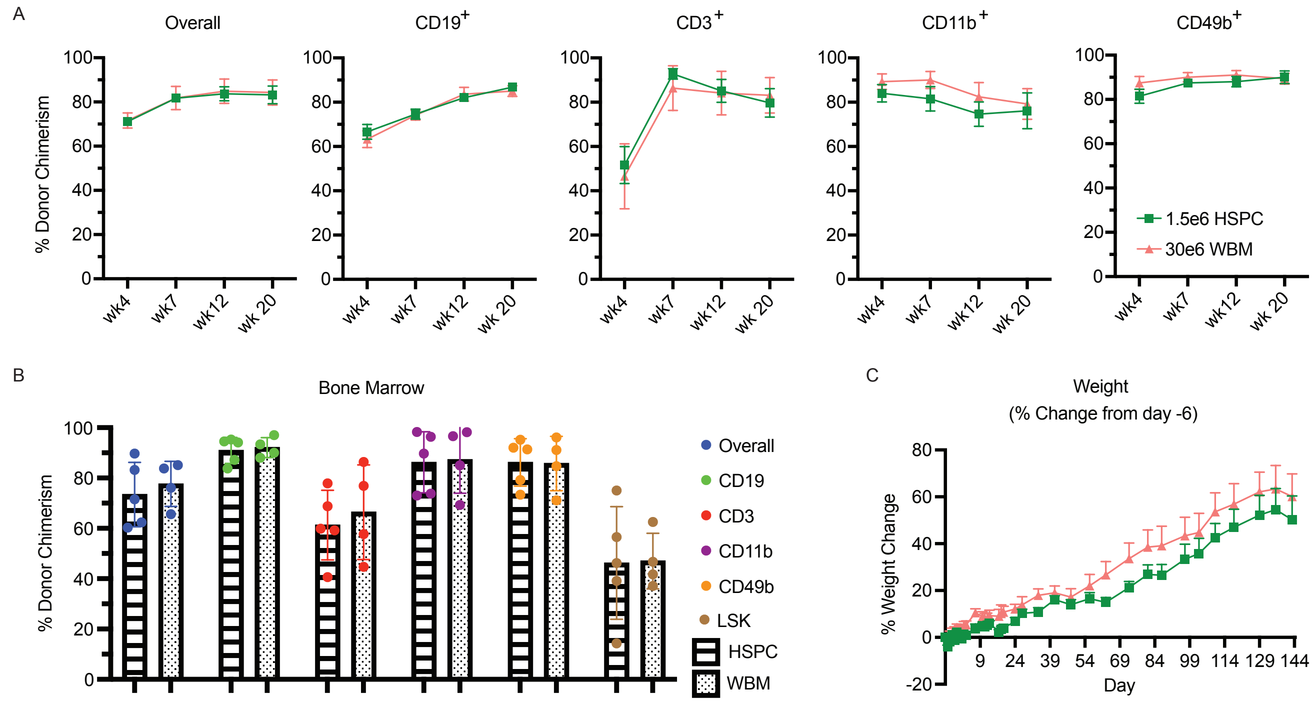

Supplementary Figure 2: HCT source material does not affect chimerism levels in conditioned B6. (A) Longitudinal multilineage chimerism analysis of peripheral blood through 20 weeks post-HCT. B6 mice were conditioned as outlined in Fig 1B and 200 cGy TBI and transplanted with 1.5e6 enriched HSPCs or 30e6 WBM cells. **(B)** Multilineage chimerism analysis, including $Lin^{-}Sca1^{+}cKit^{+}$ (LSK) HSCs, of host bone marrow 21 weeks post-HCT. (A-B) n = 5 and 4 animals for HSPC and WBM groups, respectively, from one expeirment. Data presented as mean ± SEM. HSPC = hematopoietic stem and progenitor cells; WBM = whole bone marrow.

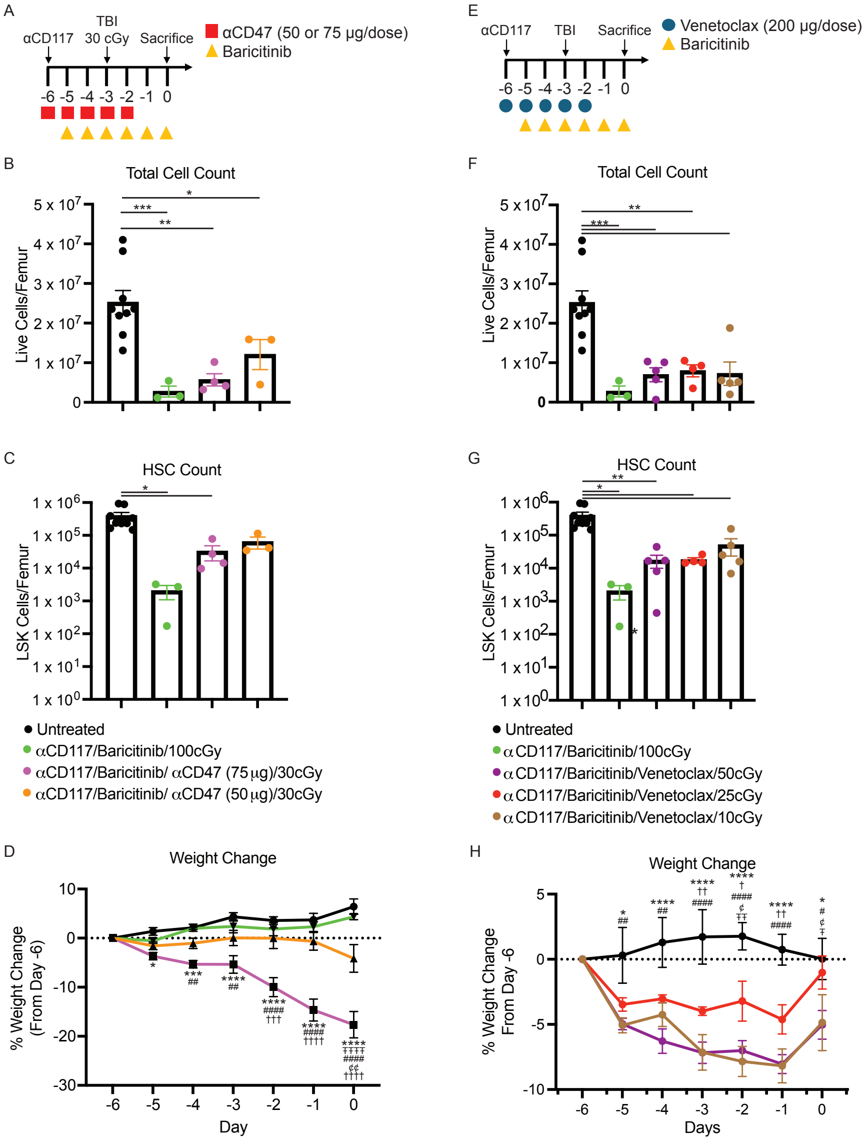

Supplementary Figure 3: Evaluation of bone marrow clearance after conditioning with aCD47 antibody and Bcl2 inhibitor. (A) Experimental conditioning timeline evaluating 5 daily doses of 50 or 75 µg/dose of aCD47 antibody. **(B)** Total live cell and **(C)** LSK cell counts per femur in unconditioned B6 CD45.1 mice and mice conditioned with αCD117/BTB/100 cGY TBI, αCD117/BTB/αCD47 (75 µg/dose)/30 cGy TBI, or αCD117/BTB/αCD47 (50 µg/dose)/30 cGy TBI. **(D)** Body weight change throughout conditioning period of as a percentage of initial weight at start of conditioning. n = 3 -9 animals from two experiments. **(E)** Experimental conditioning timeline evaluating 5 daily doses of Venetoclax and 50, 25, or 10 cGy TBI. **(F)** Total live cell and **(G)** LSK cell counts per femur in unconditioned B6 CD45.1 mice and mice conditioned with αCD117/BTB/100 cGY TBI, αCD117/BTB/venetoclax/50 cGy TBI, αCD117/BTB/venetoclax/25 cGy TBI, or αCD117/BTB/venetoclax/10 cGy TBI. **(H)** Body weight change throughout conditioning period of as a percentage of initial weight at start of conditioning. n = 3-9 animals from one to two independent experiments. (D) * indicates significance between Untreated and 75 µg αCD47; Ŧ indicates significance between Untreated and 50 µg αCD47; # signifies significance between 100 cGy and 75 µg αCD47; ¢ signifies significance between 100 cGy and 50 µg αCD47; † indicates significance between 75 and 50 µg αCD47. (H) * indicates significance between Untreated and 50 cGy; † indicates significance between untreated and 25 cGy; # signifies significance between untreated and 10 cGy; ¢ indicates significance between 50 cGy and 25 cGy; Ŧ indicates significance between 25 cGy and 10 cGy. (B, C, F, G) Data were analyzed by one-way ANOVA with Dunnett's post hoc test. (D, H) Data were analyzed by two-way ANOVA with Tukey's post hoc test. Data presented as mean ± SEM.

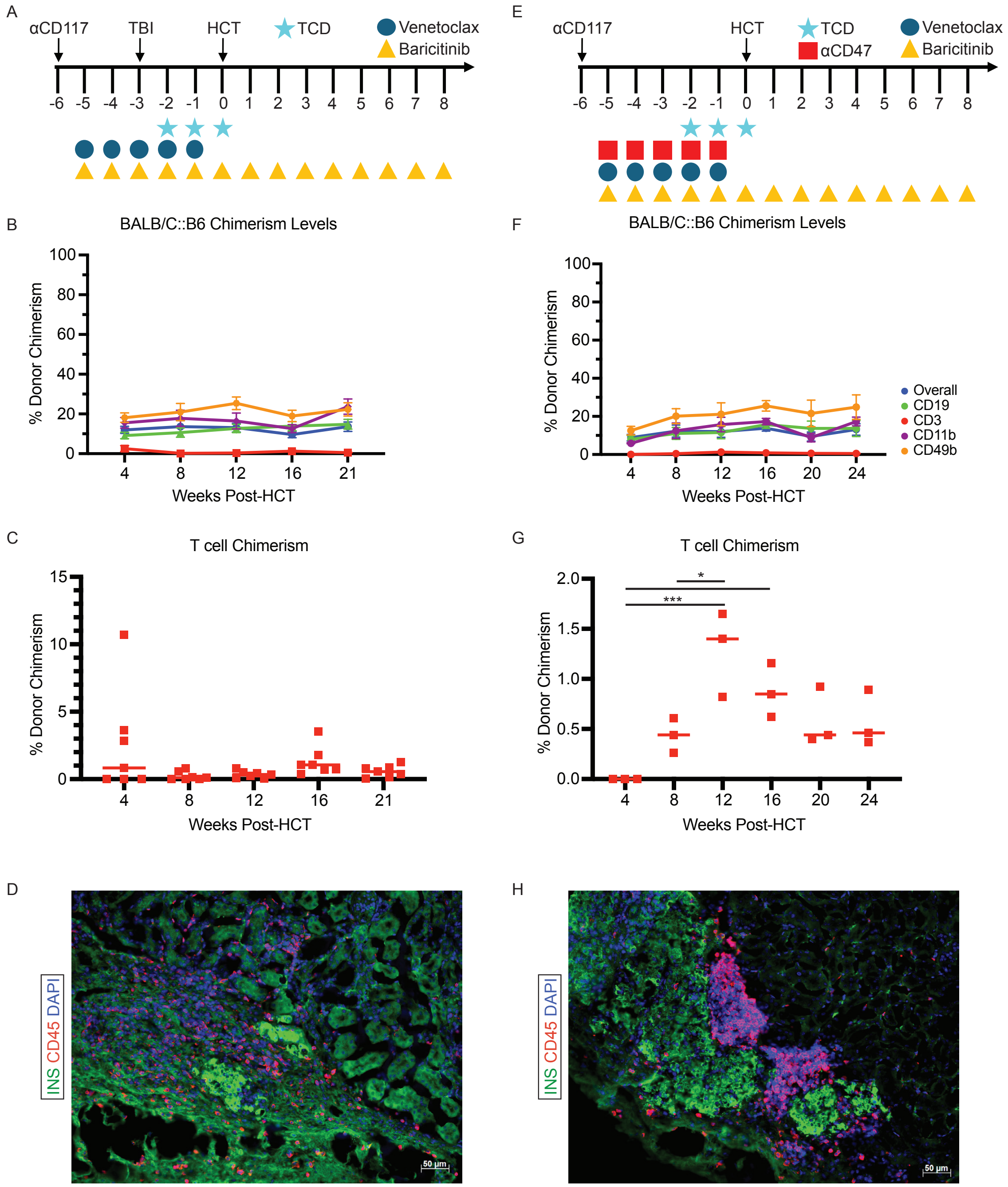

Supplementary Figure 4: Non-myeloablative conditioning requires aCD47 and 10 cGy TBI durable allogeneic donor T cell chimerism. (A) Conditioning timeline for B6 CD45.1 mice conditioned with αCD117/BTB/venetoclax/10 cGy TBI and no αCD47. **(B)** Longitudinal multilineage chimerism analysis of peripheral blood through 21 weeks post-HCT. **(C)** Individual donor T cell proportions over time. (B-C) n = 7 animals from one experiment. **(D)** Representative image of BALB/c islets transplanted under the kidney capsule of BALB/c:B6 mice stained for insulin (green) and CD45 (red). n = 3 animals from one experiment; Scale bar = 50 µm. **(E)** Conditioning timeline for B6 CD45.1 mice conditioned with αCD117/BTB/αCD47/venetoclax and *no TBI*. **(F)** Multilineage chimerism analysis of peripheral blood 4 weeks post-HCT. **(G)** Individual donor T cell proportions over time. Data were analyzed by one-way ANOVA with Tukey's post hoc test. (F-C) n = 3 animals from one experiment. **(H)** Representative image of BALB/c islets transplanted under the kidney capsule of BALB/c:B6 mice stained for insulin (green) and CD45 (red). n = 2; Scale bar = 50 µm. TBI = total body irradiation; HCT = hematopoietic cell transplant. Data presented as mean ± SEM.

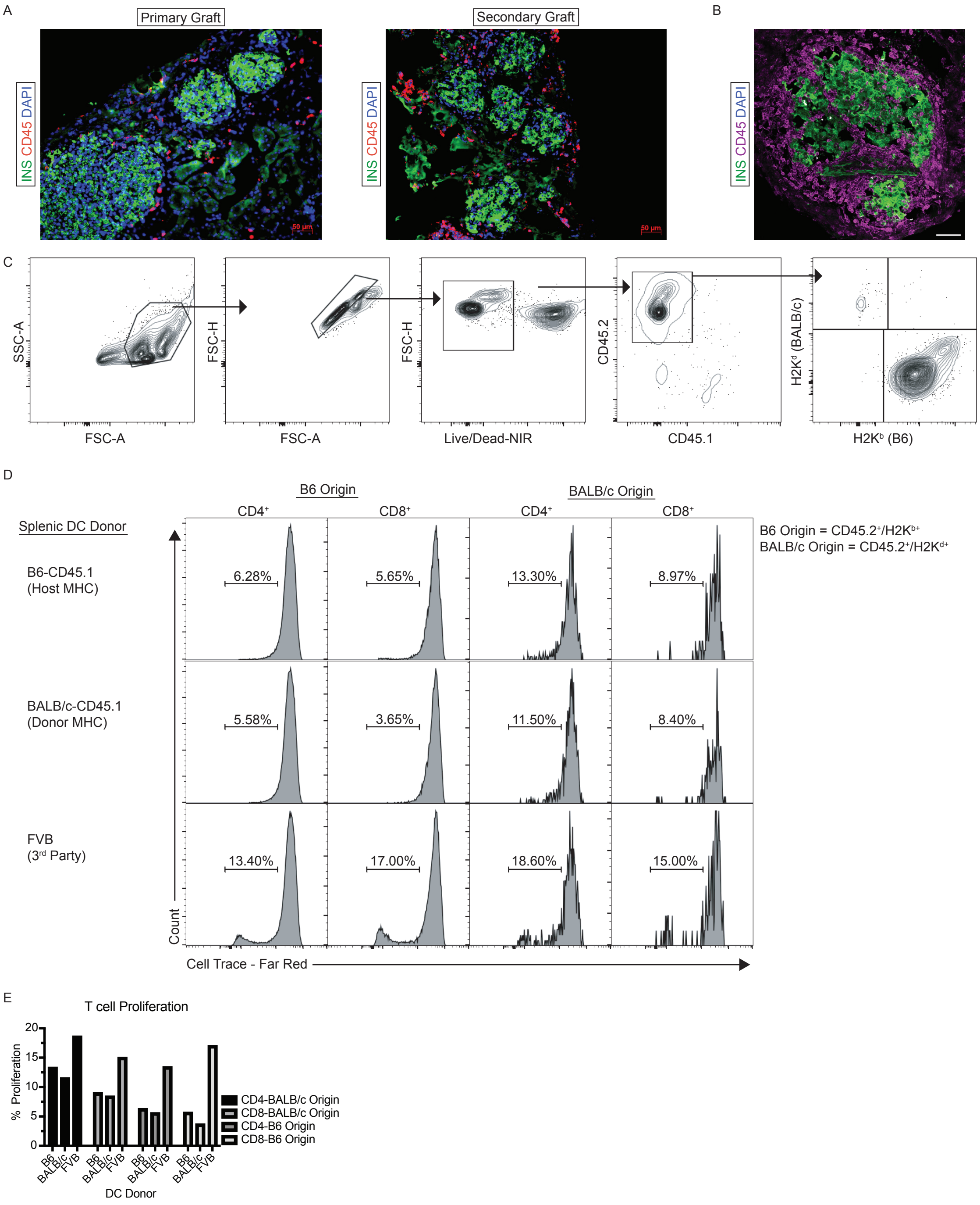

Figure S5: Sustained tolerance towards donor antigens in BALB/c:B6 chimeras: (A) Representative image of BALB/c islets transplanted under the kidney capsule of BALB/c:B6 mice stained for insulin (green) and CD45 (red). n = 2; Scale bar = 50 µm. “Primary Graft” was transplanted under the left kidney capsule on day 0 at the time of HCT, “Secondary Graft” was transplanted 51 days later under the right kidney capsule. Kidneys were harvested and assessed for immune infiltration 14 days after the secondary graft was transplanted. **(B)** Representative maximum intensity projections of third-party FVB islets transplanted under the kidney capsule of BALB/c:B6 mice stained for insulin (green), CD45 (magenta), and glucagon (white). N = 5, scale bar = 50 µm. **(C)** Gating strategy for identifying primary donor- and host-derived T cells after in vitro MLR. **(D, E)** CellTrace Far Red dilution profiles of BALB/c:B6 T cells after 72 hours co-culture with B6, BALB/c, or FVB DCs. Plots were generated by concatenating events acquired from 4 BALB/c:B6 mice, from two independent experiments, as previously described by Persaud et al (ref. 2). Triplicate wells were assessed for each group for a total of 12 technical replicates and 4 biological replicates per group.

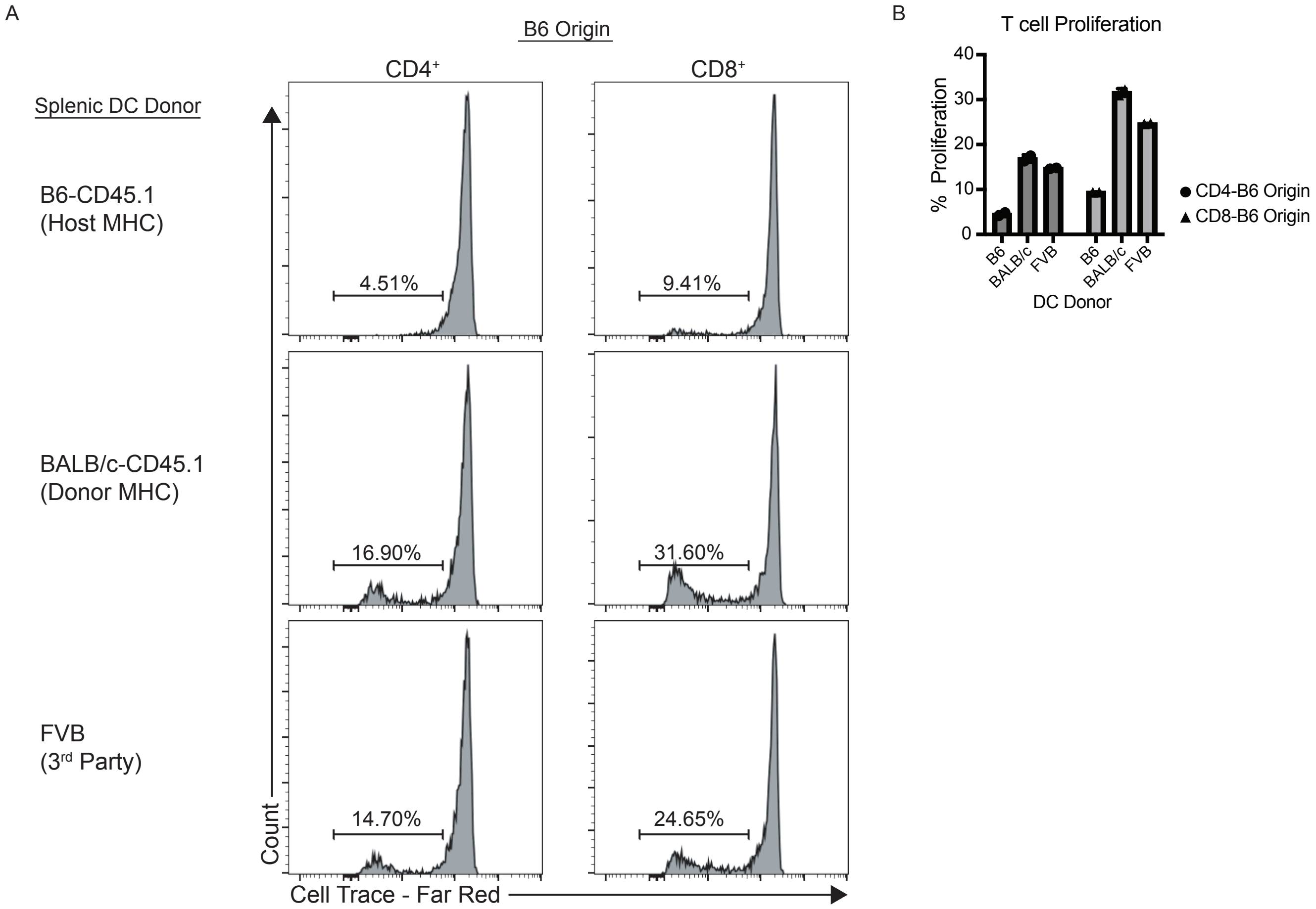

Figure S6: T cells from non-chimeric B6 mice remain responsive to BALB/c-derived antigen: (A, B) CellTrace Far Red dilution profiles of B6 T cells after 72 hours co-culture with B6, BALB/c, or FVB DCs (n=2/group).

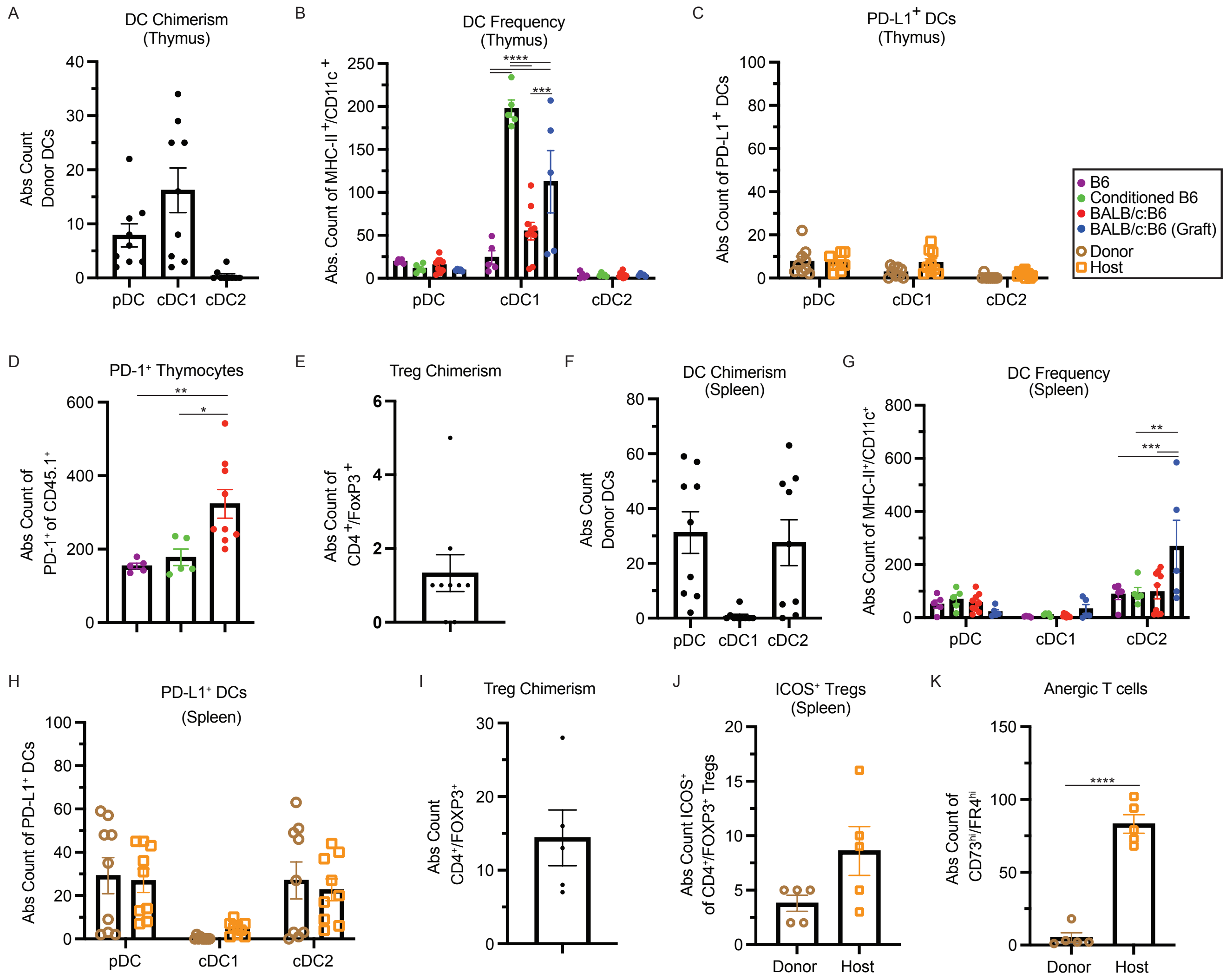

Supplemental Figure 7: Central and peripheral tolerance mechanisms in BALB/c:B6 Chimeras: Absolute cell counts of thymic and splenic DCs and Tregs. **(A-E)** Absolute cell counts corresponding to Figure 5. **(F-K)** Absolute cell counts corresponding to Figure 6. Data presented as mean ± SEM. (B, G) Data were analyzed by two-way ANOVA with Tukey's post hoc test. (D) Data were analyzed by one-way ANOVA with Tukey's post hoc test. (K) Data were analyzed by unpaired student's t test. *P < 0.05, **P < 0.01, ***P < 0.001, ****P < 0.0001.

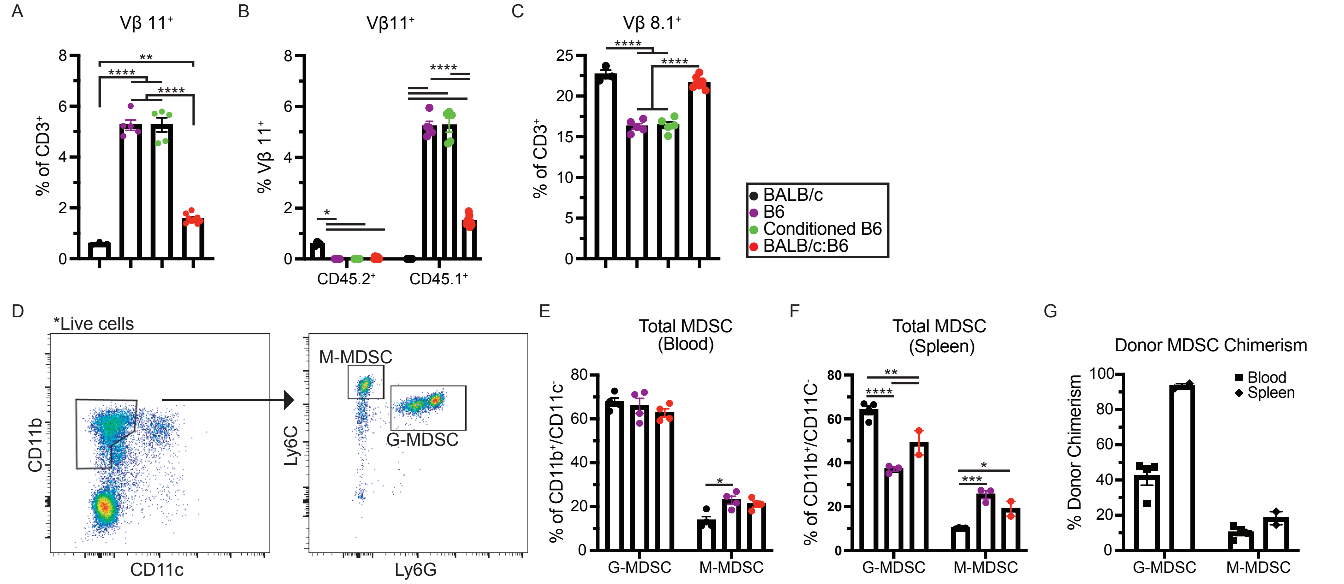

Figure S8: Deletion of donor reactive thymocytes in BALB/c:B6 mice: (A, B) $V\beta11^+$ and **(C)** $V\beta8.1^+$ splenic T cells in BALB/c, B6, conditioned B6 controls, and BALB/c:B6 mice (n = 3-9 animals from 2 independent experiments). **(D)** Gating strategy and representative flow cytometry for identifying M- and G-MDSCs in the blood and spleens of BALB/c:B6 mice. **(E)** proportion of M- and G-MDSCs of CD11c+/CD11b- cells in the **(E)** blood and **(F)** spleens of WT B6, WT BALB/c, and BALB/c:B6 mice. **(G)** Percent BALB/c donor chimerism in peripheral M- and G-MDSCs in BALB/c:B6 chimeras. (A, C) Data were analyzed by one-way ANOVA with Tukey's post hoc test. (B, E, F) Data were analyzed by two-way ANOVA with Tukey's post hoc test. Data presented as mean ± SEM. Student's t-test or one-way ANOVA was used to determine significance. *P < 0.05, **P < 0.01, ***P < 0.001, ****P < 0.0001.

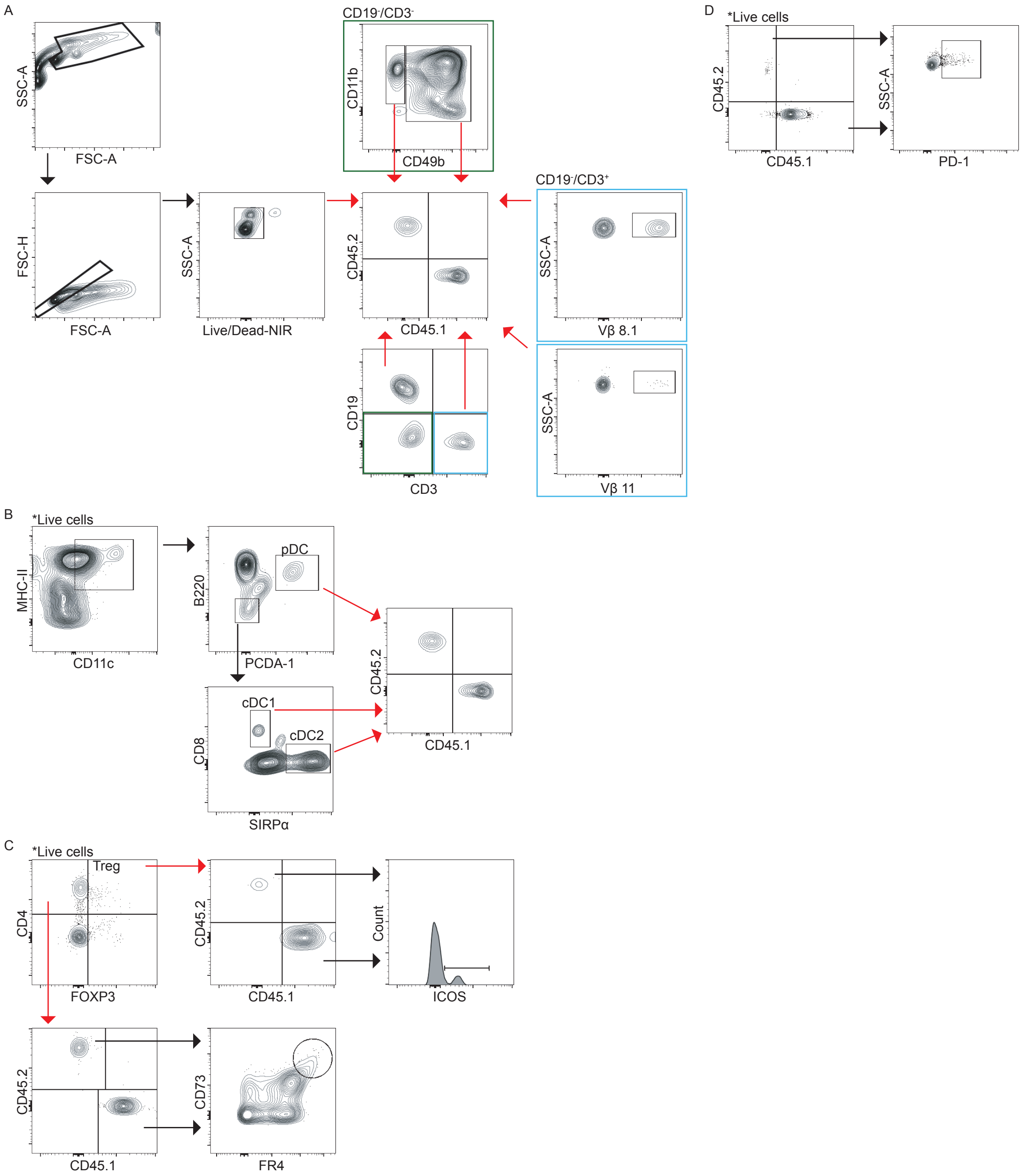

Supplementary Figure 9: Gating strategy for immune cell frequency and donor chimerism analysis: (A) Strategy for peripheral blood chimerism analysis. Donor and host cells can be differentiated by expression of CD45.2 or CD45.1, respectively. Chimerism levels of CD3$^+$ T cells, CD19$^+$ B cells, CD11b$^+$ myeloid cells, and CD49b$^+$ NK cells were assessed. **(B)** Strategy to assess dendritic cell proportions and chimerism levels in the thymus and spleen of BALB/c:B6 mice. **(C)** Strategy to assess regulatory T cell and anergic cell proportions in the thymus and spleen of BALB/c:B6 mice. Black arrows indicate gating sequence, red arrows indicate chimerism analysis. **(D)** Strategy to assess host-derived CD45.1$^+$/PD-1$^+$ thymocytes (Figure 5E).

Supplementary Table 1

				Average Chimerism (Blood)				
**Conditioning**	**Day**	**Cell Type**	**Cell Dose**	**Overall**	**B**	**T**	**Myeloid**	**NK**
**A.** Related to Figure 1 and Supplementary Figure 1								
Anti-cKit<br>TCD<br>TBI (75 cGy)<br>Baricitinib	-6<br>-2 – 0<br>-3<br>-5 – +3	HSPC	1.5e6	40.18<br>± 4.54	49.22<br>± 10.76	12.73<br>± 3.53	57.93<br>± 6.04	72.89<br>± 3.44
Anti-cKit<br>TCD<br>TBI (50 cGy)<br>Baricitinib	-6<br>-2 – 0<br>-3<br>-5 – +3	HSPC	1.5e6	14.87<br>± 3.95	16.26<br>± 3.88	0.60<br>± 0.28	24.35<br>± 3.80	38.07<br>± 7.70
Anti-cKit<br>TCD<br>TBI (25 cGy)<br>Baricitinib	-6<br>-2 – 0<br>-3<br>-5 – +3	HSPC	1.5e6	3.94<br>± 1.33	4.95<br>± 2.12	0.03<br>± 0.03	5.35<br>± 3.31	11.42<br>± 2.03
**B.** Related to Figure 2								
Anti-cKit<br>TCD<br>TBI (25 cGy)<br>Baricitinib<br>Anti-CD47 (300 µg)	-6<br>-2 – 0<br>-3<br>-5 – +3<br>-6 – -2	HSPC	1.5e6	69.74<br>± 15.51	72.29<br>± 17.49	62.13<br>± 19.16	85.67<br>± 13.90	86.43<br>± 12.29
**C.** Related to Supplemental Figure 2								
Anti-cKit<br>TCD<br>TBI (200 cGy)<br>Baricitinib	-6<br>-2 – 0<br>-3<br>-5 – +3	HSPC	1.5e6	79.96<br>± 5.96	77.53<br>± 8.83	77.32<br>± 17.94	79.09<br>± 4.43	86.74<br>± 3.69
Anti-cKit<br>TCD<br>TBI (200 cGy)<br>Baricitinib	-6<br>-2 – 0<br>-3<br>-5 – +3	WBM	30e6	80.64<br>± 6.14	76.53<br>± 10.10	75.04<br>± 19.04	85.24<br>± 5.27	89.46<br>± 1.53
**D.** Related to Figure 3 and 4								
Anti-cKit<br>TCD<br>TBI (10 cGy)<br>Baricitinib<br>Anti-CD47 (50 µg)<br>Venetoclax	-6<br>-2 – 0<br>-3<br>-5 – +8<br>-5 – -1<br>-5 – -1	WBM	75e6	27.16<br>± 2.10	28.74<br>± 2.36	5.32<br>± 3.10	40.55<br>± 4.24	48.88<br>± 3.33
**E.** Related to Supplementary Figure 3A								
Anti-cKit<br>TBI (30 cGy)<br>Baricitinib<br>Anti-CD47 (50 or 75 µg)	-6<br>-3<br>-5 – 0<br>-6 – -2	No HCT	No HCT	N/A	N/A	N/A	N/A	N/A
**F.** Related to Supplementary Figure 3E								
Anti-cKit<br>TBI (10, 25, 50 cGy)<br>Baricitinib	-6<br>-3<br>-5 – 0	No HCT	No HCT	N/A	N/A	N/A	N/A	N/A

Venetoclax	-6 – -2							
**G.** Related to Supplementary Figure 4A								
Anti-cKit<br>TCD<br>TBI (10 cGy)<br>Baricitinib<br>Venetoclax	-6<br>-2 – 0<br>-3<br>-5 – +8<br>-5 – -1	WBM	75e6	12.39<br>± 1.68	12.23<br>±2.32	1.01<br>± 0.97	17.23<br>± 4.10	21.15<br>± 2.86
**H.**	**I.** Related to Supplementary Figure 4E							
Anti-cKit<br>TCD<br>Baricitinib<br>Anti-CD47 (50 μg)<br>Venetoclax	-6<br>-2 – 0<br>-5 – +8<br>-5 – -1<br>-5 – -1	WBM	75e6	11.57<br>± 2.00	12.29<br>± 2.70	0.63<br>± 0.43	12.91<br>± 4.74	20.96<br>± 4.65

Supplementary Table 1: Conditioning regimens tested and corresponding donor chimerism levels.

Supplementary Table 2

REAGENT or RESOURCE	SOURCE	IDENTIFIER
**Antibodies**		
Goat α-Guinea Pig CF594	MilliporeSigma	Cat#: SAB4600103
Goat α-Guinea Pig CF488A	MilliporeSigma	Cat#: SAB4600040
α-Glucagon	ThermoFisher Scientific	RRID: AB_2804644<br>Cat#: PA5-88091
α-Insulin	Dako	RRID: AB_10013624<br>Cat#: A0564
α-CD45	BioLegend	RRID: AB_312966<br>Cat#: 103102
Mouse α-Rat AF488	BioLegend	RRID: AB_2910464<br>Cat#: 407513
Mouse α-Rat AF594	BioLegend	RRID: AB_2650845<br>Cat#: 407509
Mouse α-Rabbit AF594	BioLegend	RRID: AB_2832788<br>Cat#: 410407
TruStain FcX™ Antibody	BioLegend	RRID: AB_1574973<br>Cat#: 101319
α-CD45.1 BV785	BioLegend	RRID: AB_2563379<br>Cat#: 110743
α-CD45.1 PerCp-Cy5.5	BioLegend	RRID: AB_893348<br>Cat#: 110727
α-CD45.2 Pacific Blue	BioLegend	RRID: AB_492873<br>Cat#: 109819
α-CD3 AF488	BioLegend	RRID: AB_493530<br>Cat#: 100212
α-CD3 PE	BioLegend	RRID: AB_312662<br>Cat#: 100205
α-CD3 AF700	BioLegend	RRID: AB_493696<br>Cat#: 100216
α-CD4 BV421	BioLegend	RRID: AB_11219790<br>Cat#: 100544
α-CD4 PE	BioLegend	RRID: AB_313690<br>Cat#: 116005
α-CD4 AF700	BioLegend	RRID: AB_493698<br>Cat#: 100430
α-CD8a BV510	BioLegend	RRID: AB_2563057<br>Cat#: 100752
α-CD11b PE	BioLegend	RRID: AB_312790<br>Cat#: 101207
α-CD11b BV605	BioLegend	RRID: AB_11126744<br>Cat#: 101237
α-CD11c AF700	BioLegend	RRID: AB_528735<br>Cat#: 117319
α-CD19 PE-Cy7	BioLegend	RRID: AB_313654<br>Cat#: 115519

α-CD25 PE-Cy5	BioLegend	RRID: AB_312859<br>Cat#: 102010
α-CD44 BV605	BioLegend	RRID: AB_2562451<br>Cat#:103047
α-CD49b APC	BioLegend	RRID: AB_313416<br>Cat#: 108909
α-CD73 PE-Dazzle 594	BioLegend	RRID: AB_2800628<br>Cat#: 127234
α-CD172a APC	BioLegend	RRID: AB_2564060<br>Cat#: 144013
α-CD274 PE-Dazzle594	BioLegend	RRID: AB_2565638<br>Cat#: 124324
α-CD278 BV650	BioLegend	RRID: AB_2749928<br>Cat#: 313550
α-CD279 BV750	BioLegend	RRID: AB_2941421<br>Cat#: 135263
α-CD304 PE	BioLegend	RRID: AB_2561927<br>Cat#: 145203
α-CD317 PE	BioLegend	RRID: AB_1953284<br>Cat#: 127009
α-B220 FITC	BioLegend	RRID: AB_312990<br>Cat#: 103206
α-B220 PE	BioLegend	RRID: AB_312992<br>Cat#: 103207
α-FOXP3 AF647	BioLegend	RRID: AB_439749<br>Cat#: 320013
α-FR4 PE-Cy7	BioLegend	RRID: AB_1134199<br>Cat#: 125012
α-Gr-1 PE	BioLegend	RRID: AB_313372<br>Cat#: 108407
α-H2K^b PerCP-Cy5.5	BioLegend	RRID: AB_1967133<br>Cat#: 116516
α-H2K^d BV421	BioLegend	RRID: AB_2565656<br>Cat#: 116623
α-Helios AF488	BioLegend	RRID: AB_10645334<br>Cat#: 137213
α-Ly-6C BV785	BioLegend	RRID: AB_2565852<br>Cat#: 128041
α-Ly-6G Spark UV 387	BioLegend	RRID: AB_2924466<br>Cat#: 127678
α-Ter-119 PE	BioLegend	RRID: AB_313708<br>Cat#: 116207
α-Vβ8.1 FITC	BioLegend	RRID: AB_1227787<br>Cat#: 118406
α-Vβ11 PE	BioLegend	RRID: AB_10612759<br>Cat#: 139004
α-CD117 APC	eBioscience	RRID: AB_469430<br>Cat#: 17-1171-82

α-Sca-1 PE-Cy7	eBioscience	RRID: AB_469669<br>Cat#: 25-5981-82
α-CD117	BioXCell	RRID: AB_2687818<br>Cat#: BE0293
α-CD4	BioXCell	RRID: AB_1107636<br>Cat#: BE0003-1
α-CD8	BioXCell	RRID: AB_10950145<br>Cat#: BE0117
**Chemicals, Peptides, and Recombinant Proteins**		
Bovine Serum Albumin	Fisher Scientific	Cat#: BP1600-100
Cell Staining Buffer	BioLegend	Cat#: 420201
CellTrace™ Far Red Cell Proliferation Kit	Thermo Fisher Scientific	Cat#: C34564
Diphenhydramine HCl	Cayman Chemical Company	Cat#:11158
Fetal Bovine Serum	Cytiva	Cat#: SH30070.03
HBS	Caisson Labs	Cat#: HBL06
HEPES Solution	Caisson Labs	Cat#: HOL06
LANTUS®	sanofi-aventis U.S. LLC	NDC 0088-2220-33
Lineage Cell Depletion Kit, mouse	Miltenyi Biotec	Cat#:130-090-858
Liberase™ TL Research Grade	MilliporeSigma	Cat#: 05401020001
LIVE/DEAD™ Fixable Near-IR Dead Cell Stain Kit	ThermoFisher	Cat#: L34975
Penicillin-Streptomycin	Gibco	Cat#: 15140122
RBC Lysis Buffer (10X)	BioLegend	Cat#: 420301
RPMI 1640	Corning	Cat#: 10-040-CV
True-Nuclear™ Transcription Factor Buffer Set	BioLegend	Cat#: 424401
VECTASHIELD® Hard-set Mounting Medium with DAPI	Novus Biologicals	Cat#: H-1500-NB
**Experimental Models: Organisms/Strains**		
B6 CD45.1 mice	The Jackson Laboratory	Stock #: 002014
BALB/c mice	The Jackson Laboratory	Stock #: 000651
FVB mice	The Jackson Laboratory	Stock #: 001800
B6 *RIP-DTR* mice	Seung Kim Lab, Stanford University	N/A
**Software and Algorithms**		
FlowJo 10.7	FlowJo, LLC	N/A
GraphPad Prism 10	GraphPad Software	N/A
Fiji	Ref 72.	N/A
**Other**		
IC-250 X-Ray Biological Irradiator System	KIMTRON Inc	N/A
CM3050 S	Leica Biosystems	N/A
5L Aurora System	Cytek	N/A
EVOS M5000 Cell Imaging System	ThermoScientific	N/A

References

1. Parent AV, Russ HA, Khan IS, et al. Generation of functional thymic epithelium from human embryonic stem cells that supports host T cell development. *Cell Stem Cell*. 2013;13:219-229. doi:10.1016/j.stem.2013.04.004

2. Persaud SP, Yelamali AR, Ritchey JK, DiPersio JF. Conditioning with anti-CD47 and anti-CD117 plus JAK inhibition enables toxic payload-free allogeneic transplantation. *Blood Advances*. 2024;8(17):4502-4506. doi:10.1182/bloodadvances.2023012457
